# Chromosome-level genome map provides insights into diverse defense mechanisms in the medicinal fungus *Ganoderma sinense*

**DOI:** 10.1038/srep11087

**Published:** 2015-06-05

**Authors:** Yingjie Zhu, Jiang Xu, Chao Sun, Shiguo Zhou, Haibin Xu, David R. Nelson, Jun Qian, Jingyuan Song, Hongmei Luo, Li Xiang, Ying Li, Zhichao Xu, Aijia Ji, Lizhi Wang, Shanfa Lu, Alice Hayward, Wei Sun, Xiwen Li, David C. Schwartz, Yitao Wang, Shilin Chen

**Affiliations:** 1 Institute of Chinese Materia Medica, China Academy of Chinese Medical Sciences, Beijing 100700, China; 2 Institute of Medicinal Plant Development, Chinese Academy of Medical Sciences & Peking Union Medical College, Beijing 100193, China; 3 Laboratory for Molecular and Computational Genomics, Department of Chemistry, Laboratory of Genetics, UW Biotechnology Center, University of Wisconsin–Madison, Madison, Wisconsin 53706, USA; 4 Department of Microbiology, Immunology and Biochemistry, University of Tennessee Health Science Center, Memphis, Tennessee 38163, USA; 5 Queensland Alliance for Agriculture and Food Innovation, The University of Queensland, Brisbane, Australia, 4072; 6 State Key Laboratory of Quality Research in Chinese Medicine, Institute of Chinese Medical Sciences, University of Macau, Macau, 999078, China

## Abstract

Fungi have evolved powerful genomic and chemical defense systems to protect themselves against genetic destabilization and other organisms. However, the precise molecular basis involved in fungal defense remain largely unknown in Basidiomycetes. Here the complete genome sequence, as well as DNA methylation patterns and small RNA transcriptomes, was analyzed to provide a holistic overview of secondary metabolism and defense processes in the model medicinal fungus, *Ganoderma sinense*. We reported the 48.96 Mb genome sequence of *G. sinense*, consisting of 12 chromosomes and encoding 15,688 genes. More than thirty gene clusters involved in the biosynthesis of secondary metabolites, as well as a large array of genes responsible for their transport and regulation were highlighted. In addition, components of genome defense mechanisms, namely repeat-induced point mutation (RIP), DNA methylation and small RNA-mediated gene silencing, were revealed in *G. sinense*. Systematic bioinformatic investigation of the genome and methylome suggested that RIP and DNA methylation combinatorially maintain *G. sinense* genome stability by inactivating invasive genetic material and transposable elements. The elucidation of the *G. sinense* genome and epigenome provides an unparalleled opportunity to advance our understanding of secondary metabolism and fungal defense mechanisms.

*Ganoderma*, which is a macrofungus in Basidiomycetes, is well known as a medicinal fungus in Asia, where it has been used to treat various diseases for more than 2000 years. Their medicinal properties are considered as a result of a rich diversity of secondary metabolites, including sterols, alkaloids and terpenoids. Recently, the genome of *G. lucidum* and *G. sp.* have been sequenced for the study of the molecular mechanisms of secondary metabolism and lignin degradation^1^,^2^.

As a saprophytic fungus living in complex ecological niches, *Ganoderma* requires a series of powerful defense systems to protect itself from the threat of competitors and invaders. Genome defense is one of the major fungal defense systems developed to protect the genomes of the organisms from invasion by foreign genetic material and transposable elements (TEs), thus maintaining genome stability^3^. Repeat-induced point mutation (RIP) was the first reported fungal genome defense system and was discovered in *Neurospora crassa*^4^. This mechanism specifically introduces cytosine-to-thymine transitions in repetitive sequences to limit the movement of selfish DNA or mobile DNA during meiosis^4^,^5^,^6^,^7^. DNA methylation is an ancient epigenetic DNA regulatory mechanism that is widely involved in gene silencing in eukaryotes, and this mechanism also plays an important role in fungal genome defense^8^,^9^. The methylated component of the *N. crassa* genome consists almost exclusively of relics of transposable elements that were subject to RIP^10^. Two additional genetic mechanisms, quelling and meiotic silencing by unpaired DNA (MSUD), have been identified and have been shown to be associated with small RNA-mediated genome defense^11^,^12^,^13^.

Fungi are a rich and sustainable source of secondary metabolites. Although the ecological role of most fungal secondary metabolites is still obscure, a growing body of evidence has demonstrated that chemical defense strategies are commonly used by fungi in response to biotic and abiotic environments, including defense against herbivores and fungivores^14^. A variety of fungal secondary metabolites are involved in chemical defense processes, including alkaloids, polyketides and terpenoids^15^. In filamentous fungi, genes required for the biosynthesis of a specific secondary metabolite are generally clustered together and are often located near telomeres^16^. A classic fungal gene cluster contains genes encoding the signature enzymes that synthesize the skeletons of the different classes of secondary metabolites, such as non-ribosomal peptide synthases (NRPSs), polyketide synthases (PKSs) and terpenoid synthases (TSs). These clusters also contain genes encoding tailoring enzymes that modify the skeletons, such as oxidoreductases, methyltransferases and glycosyltransferases^17^.

In this work, we assembled a high quality genome of *G. sinense* and compared them with the other two sequenced genomes of *Ganoderma* species. We then examined whole-genome DNA methylation patterns and sequenced the small RNA transcriptome to provide a holistic overview of the genome defense processes in *G. sinense*. We also identified a large array of genes involved in the biosynthesis of secondary metabolites and their regulation. These results will ultimately lead to a better understanding of the roles of specific genes and regulatory pathways in chemical defense systems, thereby presenting a novel avenue for drug discovery.

## Results

### Genome Sequence and Annotation

**Figure 1:**
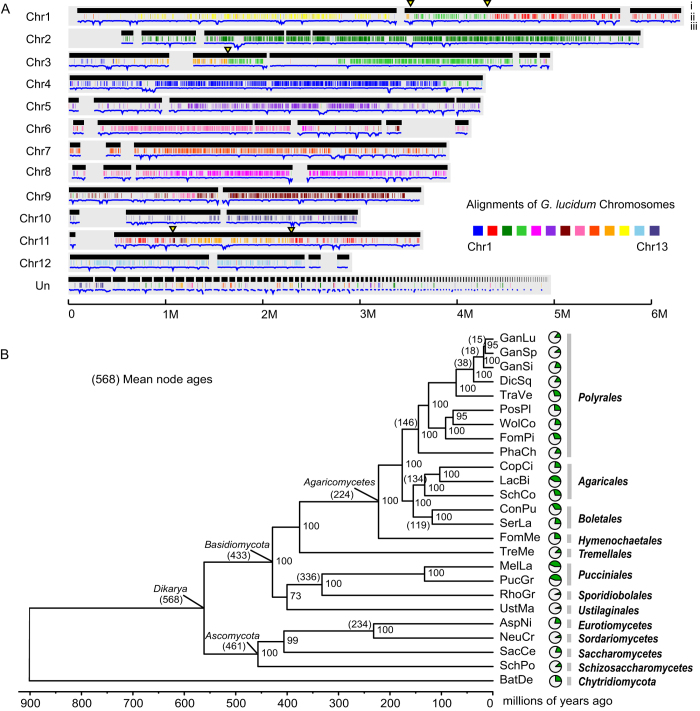
Genome comparison and phylogenetic relationships. **A**) The *G. sinense* genome was aligned, as the reference genome, with the *G. lucidum* genome. The grey background represents the 12 chromosomes. For each chromosome, row i represents the location of the *G. sinense* scaffolds on each chromosome, and row ii represents the fragments of the *G. lucidum* genome, which were mapped using MUMmer. The breakpoints are labeled as yellow triangles. Row iii represents the distribution of the transposable elements. Chr, chromosome. **B**) Phylogenetic relationships of *Ganoderma* and other fungi. A concatenated alignment of 59 single copy genes from 25 fungi was used to construct a phylogenetic tree using the maximum likelihood method. The mean node age was labeled using parentheses. After each node, bootstrap values higher than 50 were marked. The pie graphs exhibit the proportion of paralogous genes for each species. Full name of species were shown in methods.

The genome of *G. sinense* (strain ZZ0214-1) was shotgun sequenced to ~500x coverage using a combination of next-generation sequencing technologies (see Supplementary Table S1). The sequences were assembled into 69 scaffolds with a total length of 48.96 Mb, and the assembly represents the largest genome of the three *Ganoderma* species sequenced to date (see Table 1). Approximately 94% of the assembled genome was distributed over 42 scaffolds that were ordered and anchored onto the optical map scaffolds of the 12 *G. sinense* chromosomes constructed using optical mapping technology^18^,^19^ (see Fig. 1A), suggesting the high quality of the genome sequence assembly. The average GC content of the *G. sinense* genome (55.59%) was comparable to that of the *G. lucidum* genome (55.9%)^1^, but it was higher than that of other sequenced Basidiomycota fungi (average of 50.63%) (see Supplementary Fig. S1).

A total of 15,688 gene models were predicted, of which 87% were supported by the assembled RNA-Seq transcripts. In total, 85% of the genes were annotated by searching against public databases, with 65% of the annotated genes containing Pfam domains. Additionally, 202 tRNAs, including nine pseudogenes, were predicted, with an average length of 82 bp.

Transposable elements (TEs), including retrotransposons and DNA transposons, accounted for approximately 12.2% of the *G. sinense* genome. RNA-mediated retrotransposons were more abundant than DNA-mediated transposons (see Supplementary Table S2), consistent with other *Ganoderma* species. Long terminal repeats (LTRs) were the most abundant class of retrotransposons, among which the *Gypsy* and *Copia* were the largest subclasses. In *G. sinense*, long interspersed nuclear elements (LINEs) were much more abundant than in the other two fungi (see Supplementary Table S2). DNA transposons accounted for 2.2% of the *G. sinense* genome, and these transposons were primarily comprised of terminal inverted repeats (TIRs), *helitrons* and miniature inverted-repeat transposable elements (MITEs).

### Genome Comparison and Phylogenetic Relationships

Orthology analysis was carried out using the genome sequence data for the three sequenced *Ganoderma* species (*G. sinense*, *G. lucidum* and *Ganoderma sp.*) and 22 other fungi from Basidiomycota, Ascomycota and Chytridiomycota (see Supplementary Table S3). Approximately three-quarters of the genes in *G. sinense* were included in a total of 9,336 orthologous groups. The *G. sinense* genome was found to contain more paralogous genes than the other two *Ganoderma* species (see Fig. 1B). A total of 3,122 paralogous genes were identified, accounting for 19.9% of all the *G. sinense* genes. Almost all of these genes were found in orthologous groups, suggesting that the paralogs of *G. sinense* are primarily derived from ancient gene duplication events.

Fifty-nine single-copy genes identified in the orthology analysis were used to construct a phylogenetic tree using the maximum likelihood method (see Fig. 1B). The tree topology was generally consistent with prior analyses^20^. Of the available sequenced genomes, *Dichomitus squalens* was the closest relative of the three *Ganoderma* species in the Polyrales clade. In addition to *Trametes versicolor*, these fungi formed a clade of five white-rot fungi. Some of the major medicinal fungal species used in China, including *Ganoderma*, *T. versicolor* and *Wolfiporia cocos*, belonged to the Polyrales clade, suggesting that this order may be a source of medicinal fungi for drug development. Based on fossil calibrations at the three nodes, including the ancestors of Boletales, Agaricales and Ascomycota, the divergence time of *Ganoderma* and *Dichomitus* was estimated to be approximately 38 Ma (millions of years ago), with *G. sinense* appearing at approximately 18 Ma.

A whole-genome comparison between *G. sinense* and *G. lucidum* using the MUMmer software revealed similar chromosome organization, with ~80% similarity between the alignment blocks (see Fig. 1A and Supplementary Fig. S2). Five major breakpoints were found between *G. sinense* and *G. lucidum*, suggesting species-specific chromosomal rearrangements (see Fig. 1A). Synteny analysis revealed 160 syntenic blocks comprised of 7,524 genes and 7,509 genes in *G. sinense* and *G. lucidum*, respectively. Additionally, 115 syntenic blocks were identified between *G. sinense* and *G. sp.*, comprised of 7,623 and 7,539 genes, respectively. A total of 5,729 homologous genes in *G. sinense* were not syntenic with *G. lucidum* or *G. sp*., suggesting a number of gene rearrangement events during *Ganoderma* evolution.

### Genome Wide DNA Methylation

**Figure 2:**
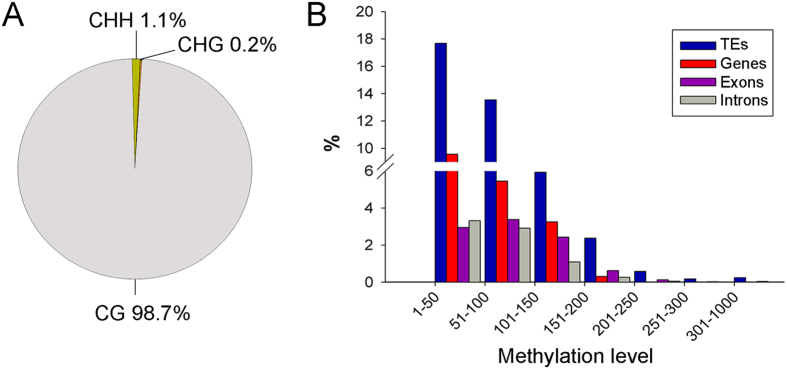
The characteristics of cytosine methylation. **A**) Characteristics of the dinucleotide and trinucleotide contexts of the methylation loci. **B**) Comparison of the methylation levels among the TEs, genes, exons and introns.

Whole-genome bisulfite sequencing was used to detect 5-methylcytosine (5mC) in *G. sinense*. Approximately 1.8% of the cytosine residues were methylated in the *G. sinense* genome, among which 98.7% were specific to CpG dinucleotides (see Fig. 2A). To ascertain a possible contextual bias of DNA methylation, the methylation level (number of methylated cytosines per kilobase) of the TEs versus the coding regions was determined. As shown in Fig. 2B, the methylation level in the TE regions was higher than in the genic regions, which is consistent with results observed in other fungal genomes^9^, and up to 33% of the CpG dinucleotides were subject to methylation.

DNA methylation is catalyzed by DNA methyltransferase (DMTase), which transfers the methyl group from a methyl donor onto a cytosine. Phylogenetic analysis of the DMTase sequences suggested that *G. sinense* has at least three DMTases (see Supplementary Fig. S3). These genes were found to be homologous to *Dnmt1a* (46% similarity with *Postia placenta*), *Dnmt1b* (36% with *P. placenta*) and *Dnmt2* (53% with *Coprinopsis cinerea*) (see Supplementary Table S4 and S5). Domain analysis showed that these DMTases have a relatively consistent domain structure within the same subfamily (see Supplementary Table S6 and Supplementary Fig. S4).

Trimethylation of histone H3 on lysine 9 (H3K9me3) by an H3-specific histone methyltransferase (HMTase) is essential for DNA methylation. An H3K9-specific HMTase homolog was found in *G. sinense*, and this protein contained three specific domains: pre-SET, SET and post-SET (see Supplementary Fig. S4). Additionally, putative accessory proteins for H3K9me3 and cytosine methylation were found, including CUL4 (Cullin 4), DDB1 (Damaged DNA Binding Protein-1) and HP1 (Heterochromatin Protein 1) (see Supplementary Table S5).

### Repeat-Induced Point Mutation

**Figure 3:**
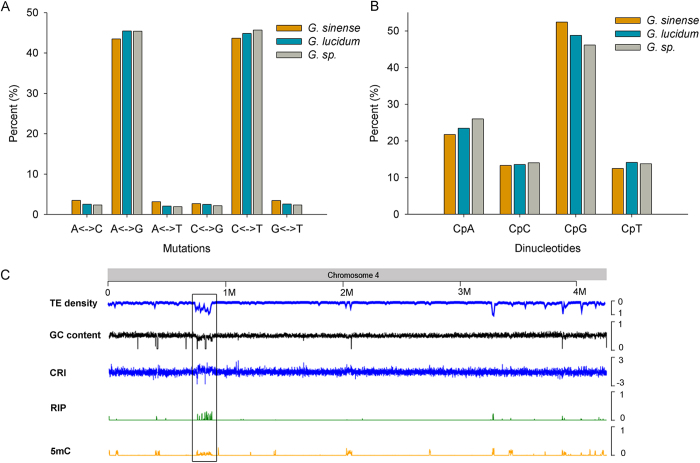
The characteristics of the RIP mutations. **A**) The mutation types investigated in the TEs in *G. sinense*, *G. lucidum* and *G. sp*. **B**) The dinucleotide patterns for RIP. The frequencies of dinucleotides in each CpN dinucleotide were assessed in *G. sinense*, *G. lucidum* and *G. sp*. **C**) The enrichment of RIP and 5 mC on chromosome 4. The density of TEs is shown at the top of the graph. The base composition (GC%), a composite RIP index (CRI), plotted in moving 500 bp windows with 100 bp steps, are shown. The RIP and 5 mC, also calculated in the above windows, are shown with their densities for each window at the bottom.

Previous RIP investigations mainly focused on Ascomycetes; however, a recent study suggested the existence of RIP in Basidiomycetes^21^. To determine whether RIP exists in *G. sinense*, TEs with a minimum length of 400 bp and a sequence identity greater than 80% were analyzed for RIP hallmarks using a sequence alignment-based algorithm^6^,^22^. Within these repetitive regions, transitions from C:G to T:A accounted for more than 87% of all point mutations (see Fig. 3A), suggesting the existence of RIP in *G. sinense*. Similarly, *G. lucidum* and *G. sp.* also have RIP processes, as suggested by their high percentages of RIP-like transition mutations (90% and 91%, respectively; see Fig. 3A). RIP is not random, and CpN dinucleotides are preferentially altered in *G. sinense*. Quantification of the dinucleotide frequency in the RIP-affected sequences revealed a preference for CpG dinucleotides in *Ganoderma* species, compared with the CpA dinucleotides observed in *N. crassa* (see Fig. 3B).

To understand the possible coordination between cytosine methylation and RIP in TE silencing in *G. sinense*, we investigated the methylation level of the RIP-affected TEs and found that almost all of these TEs were methylated. In addition, the distribution of RIP and DNA methylation showed a direct relationship. At least 10 RIP and methylation-rich regions were discovered in the genome based on the following four defining characteristics: relatively low GC density, high composite RIP index (CRI)^23^ and enrichment of RIP and methylation (Refer to Fig. 3C for an example).

### Small RNA-mediated Silencing

**Figure 4:**
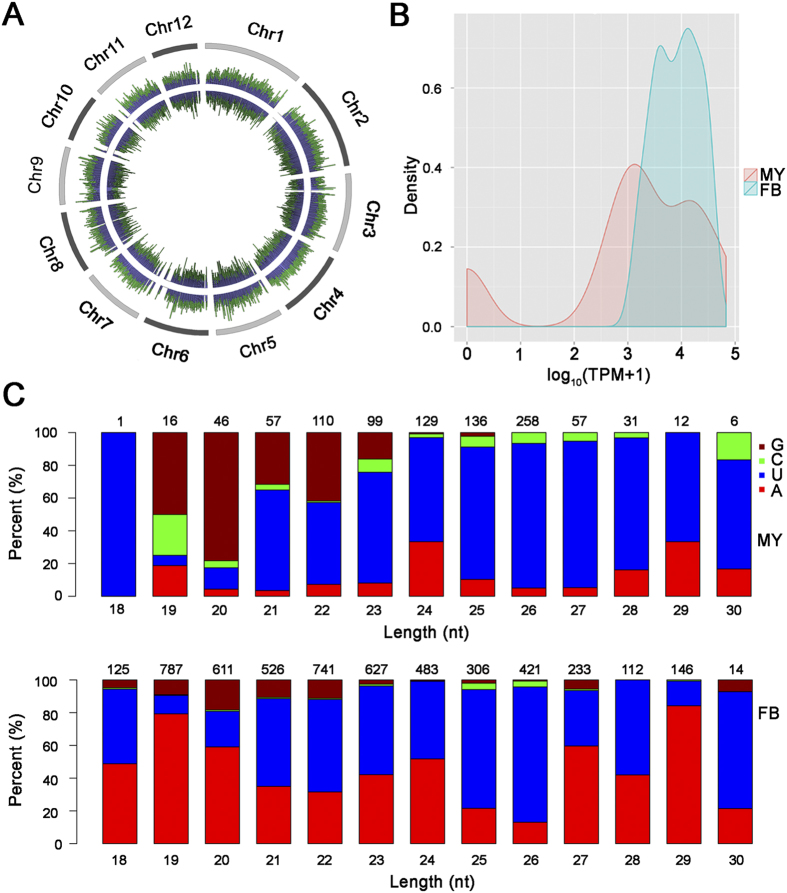
Characterization of small RNAs in *G. sinense*. **A**) Distribution of small RNAs mapping to *G. sinense* chromosomes. From outside to inside, grey lines represent chromosomes. Line graph represents forward and reverse mapped small RNAs. Green and purple lines represent the density of small RNAs in mycelia and fruiting bodies, respectively. **B**) Distribution of small RNA TPM (transcripts per million clean reads) values in *G. sinense* mycelia (MY) and fruiting bodies (FB). **C**) Nucleotide bias at the 5’-terminus of small RNAs in *G. sinense* mycelia (MY) and fruiting bodies (FB).

To unravel the potential roles of RNA silencing in *G. sinense* genome defense, two different small RNA (smRNA) libraries were generated from mycelium and fruiting bodies in *G. sinense* and then subjected to Illumina high-throughput sequencing. To identify small RNAs specific to mycelium and fruiting bodies, total reads were grouped into 1,655,536 unique sequences. Only 65,362 (3.95%) unique sequences were common to both tissues. Of the remainder, most sequences (1,035,653; 62.56%) were specific for fruiting bodies, while 33.49% (554,521) were specific to mycelium. Sequences with length 18–30 nt were mapped to the haploid *G. sinense* genome, generating 1,812,452 (76%) and 5,705,067 (89%) genome-matched reads from mycelium and fruiting bodies, respectively (see Fig. 4A). A large number of these mapped reads were annotated as non-coding rRNA (34.04% and 53.78%), and as tRNA (11.13% and 1.51%). Only a small number of mapped reads were classified as milRNA (958 and 5,132) (see Fig. 4B) and disiRNA (594 and 10,027).

Precursors of milRNA can produce stable hairpin structures^24^. On the basis of this characteristic, sixty-three novel milRNAs (18–25 nt) were identified in *G. sinense* (see Supplementary Table S7). Analysis of 5’-terminal nucleotide bias revealed that uracil (U) was the most frequent 5’-terminal nucleotide in milRNAs of *G. sinense*, while the frequency of adenine (A) significantly increased in fruiting bodies (see Fig. 4C).

Analysis of milRNA targets showed possible regulation of a variety of transport process. Two species of milRNAs were targeted to two members of the major facilitator superfamily (MFS), involved in transmembrane transport. milRNA-4 was predicted to act on the MFS gene *GS04616*. Meanwhile, milRNA-19 was predicted to target the MFS gene *GS13257*, whose expression correlated to milRNA-19 abundance. In mycelia, *milRNA-19* was silenced and *GS13257* was highly expressed, whereas in fruiting bodies, *milRNA-19* was upregulated and *GS13257* was significantly downregulated. Thus milRNA-19 may silence the expression of *GS13257* and inhibit it’s specific function in transmembrane transport.

In *G. sinense*, more than 40 regions of the genome were found where both strands of the DNA were almost equally mapped by strand-specific smRNA reads, suggesting that both genomic DNA strands are transcribed^24^ (see Supplementary Table S8). Therefore, double-stranded RNA formed from these regions may be further processed into disiRNAs^24^. Seventeen of these regions were located at the ends of chromosomes, presumably close to the subtelomeric regions, which are particularly rich in TEs (see Supplementary Table S8).

In *G. sinense*, a series of complex components involved in quelling and meiotic silencing by unpaired DNA (MSUD) were discovered including candidate genes encoding the RNA-dependent RNA polymerases (RdRPs), Dicer and Argonaute proteins, using funRNA and functional annotation methods^12^,^25^ (see Supplementary Table S9). Some pathway-specific genes were also found, including two *qde-1*-like genes specific for quelling and a *sad-1*-like gene specific to the MSUD pathway. These findings suggest that small RNA-mediated genome defense mechanisms are most likely conserved in this fungus.

### TE Inactivation by Genome Defense

Class I TEs, also known as retrotransposons, are mobilized in genomes through the action of their RNA intermediates. Therefore, the expression of class I TEs provides direct evidence for their activity. By mapping the transcripts from the mycelium to the TE sequences, we classified approximately one third of the class I TEs as active during this developmental stage (see Table 2). Among the remaining inactive class I TEs, 63% were affected by RIP mutations or DNA methylation. Silencing of the remaining 37% of the inactive class I TEs may be caused by RNAi, indel mutations during translocation or other unknown mechanisms. In total, approximately 93.2% of the class I TEs subjected to both RIP mutations and DNA methylation were silenced, suggesting that the strong silencing was induced by the dual effects of RIP and DNA methylation.

Unlike class I TEs, class II TEs, also known as DNA transposons, move throughout the genome using a “cut and paste” mechanism, without the need for RNA intermediates. Transposases are essential for this process. Although some nonautonomous DNA transposons do not contain transposase genes, transposases encoded by other autonomous transposons may help carry out their movement. Therefore, it is very difficult to estimate DNA transposon activity by analyzing their transcript levels. In total, 110 transposase genes were found in the *G. sinense* genome, and these genes were assigned to the TIR, MAVERICK and *helitron* superfamilies. Within the TIR superfamily, 93 members were classified as CACTA, *Tc1/Mariner*, *PIF/Harbinger*, *P* and *hAT* elements. Twenty-five transposases had potential nucleic acid binding activity (GO:0003676) or DNA binding activity (GO:0003677). Eighty-six transposase genes were methylated, and only 11 of these were expressed; therefore, DNA methylation may inactivate the class II TEs by silencing the transposase genes.

### Gene Clusters Involved in Biosynthesis of Secondary Metabolites

**Figure 5:**
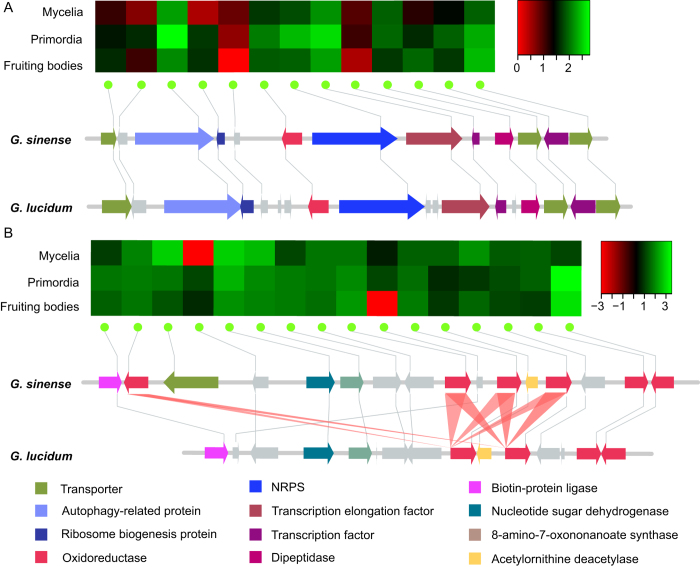
Secondary metabolism gene clusters and their expression. **A**) NRPS gene clusters and their expression profiles across three different developmental stages: mycelia, primordia and fruiting bodies. The heatmap shows the Log_10_ (RPKM) values as colors increasing from red to black to green. The different colors in the gene clusters of *G. sinense* and *G. lucidum* represent the different types of genes. **B**) CYP gene clusters and their expression profiles. The red triangles connect the CYP5139 homologous genes between *G. sinense* and *G. lucidum*.

As an important medicinal fungus, *G. sinense* produces a variety of secondary metabolites including steroids, alkaloids and terpenoids^26^,^27^,^28^,^29^, many of which are most likely involved in chemical defense. In most fungi, the genes related to the biosynthesis of secondary metabolites are generally clustered together. In *G. sinense*, one non-ribosomal peptide synthetase (NRPS), six polyketide synthases (PKSs), seven NRPS-like genes and twenty terpene synthases were located in gene clusters predicted using the antiSMASH and/or SMURF software^30^,^31^. Among these putative clusters, one NRPS cluster had a similar organization to that found in *G. lucidum*, which contains multiple genes encoding tailoring enzymes, transcription factors and transporters, as well as the signature NRPS gene (see Fig. 5A). Five genes in this putative cluster were co-expressed with NRPS during fungal development with a correlation coefficient of >0.9, which is consistent with the existence of a gene cluster organized around the NRPS gene. A 310-kb region of chromosome 7 contained four PKSs and a 150-kb TE-enriched fragment with varying DNA methylation and transcriptional gene silencing profiles. Most of the genes were collinear with *G. lucidum*, except for those in the TE-enriched region, indicating the conserved gene order in the non-TE regions. The origin of the TEs and duplicated PKSs should be further investigated.

### CYP Gene Clusters and Heavily Methylated CYPs

Triterpenoids are derivatives of lanosterol that comprise a major class of bioactive compounds in *G. sinense*. All eleven enzymes in the biosynthetic pathway of lanosterol were identified in this fungus (see Supplementary Table S10). Cytochrome P450s (CYPs) greatly contribute to the diversity of triterpenoids through a series of oxidation reactions^32^,^33^. In our study, a total of 237 CYPs, including nine pseudogenes, were identified and classified into 41 families. The CYP distribution in the genomes of *G. sinense* and *G. lucidum* was very different, with only 11 CYPs found in the identified syntenic regions between these two species. Compared with *G. lucidum*^1^, *G. sinense* was missing the CYP5363 family, while the number of CYP512s and CYP5035s was greatly increased (see Supplementary Table S11). A total of 44 CYPs were located in the gene clusters shown to contain secondary metabolite biosynthesis genes. At least 68 tandemly-duplicated CYP genes were found to belong to 29 physical clusters, and each cluster was comprised of the same CYP gene family. This suggests that a number of gene duplication events contributed to the expansion of the CYP gene families in *G. sinense*. An example of CYP evolution by duplication is presented in Fig. 5B. Here, a tandem CYP cluster contains four CYP5139Gs and two CYP5359s, while the corresponding cluster in *G. lucidum* contains only two CYP5139Gs and two CYP5359s, indicating the species-specific CYP5139 gene duplication following the divergence of these two species (see Fig. 5B).

However, these CYPs were not always functionalized, in sometimes, they may be regulated by specific mechanisms, such as DNA methylation. In our study, at least 99 CYPs were located in the TE-rich regions, suggesting that some CYP gene duplications and rearrangements may be associated with TE translocation. Seven of the CYP genes found in the TE-rich regions were heavily methylated, and these genes belonged to three expanded CYP families: CYP5035, CYP5150 and CYP5359. Of the methylated CYP5150 genes, three (CYP5150D27a-c) genes showed more than 90% similarity and were adjacent to each other (see Supplementary Table S12). Alignment of these gene sequences suggested that a tandem duplication event (indicated as event I), covering two 10-kb regions (see Supplementary Fig. S5), may have occurred, and this event may have resulted from an unequal crossing over event during meiosis due to the close proximity and high sequence similarity. CYP5150D27c was located outside of these regions; thus an additional single gene-specific duplication event (indicated as event IV) may have occurred.

### Regulation and Transport of Secondary Metabolites Regulated by DNA Methylation

Fungal secondary metabolism is controlled by a complex regulatory network involving multiple genetic, epigenetic and proteomic control points^17^,^34^. Transcription factors (TFs) play a central role in regulating pathway flux and in controlling the expression of key pathway enzymes. In total, 449 transcription factors were identified in the *G. sinense* genome, and these genes were classified into 48 families (see Supplementary Table S13). Among these, at least 29 TF genes were located in secondary metabolism gene clusters, suggesting that they may encode pathway-specific TFs.

At least 74 TF genes were methylated in the fungal mycelia. Most genes belonged to the CCHC-type and Cys_2_His_2_-type zinc finger TF families and were located in the TE-rich regions. RNA-Seq revealed that 93% of the methylated TF genes were silenced. Of the 29 TF genes located in secondary metabolism gene clusters, seven were methylated and five of these genes were silenced during development. Consequently, it is conceivable that secondary metabolism gene clusters and their associated transcription factors are regulated by DNA methylation in *G. sinense*.

Fungal growth conditions in culture, including the carbon and nitrogen source, mineral ion complement, light intensity, pH and oxygen supply^17^, can greatly influence the biosynthesis of secondary metabolites. Global TFs are involved in the response to environmental signals^17^. In *G. sinense*, two Cys2His2-type zinc finger TFs, CreA and PacC, were identified, which control the response to the carbon source^35^,^36^ and pH signals^37^, respectively. A GATA family TF, AreA, involved in the nitrogen response^38^, as well as components of the Velvet complex, VeA^39^ and VelB^40^, were also found. The Velvet complex generally regulates the cross-talk between the fungal developmental and secondary metabolism^40^.

In *G. sinense*, seven classes of transporters were identified (see Supplementary Table S14), some of which play essential roles in the transport of secondary metabolites across cell membranes^41^. Two major families are involved in the transport of small secondary metabolite molecules, including 235 major facilitator superfamily (MFS) and 53 ATP-binding Cassette (ABC)^42^,^43^. A total of 112 transporters were located in secondary metabolism gene clusters, suggesting their possible roles in the transport of specific secondary metabolites. Thirty-six of these transporters were predominantly methylated and transcriptionally silenced, suggesting that methylation-induced transcriptional gene silencing may be involved in the regulation of secondary metabolite transport in this fungus.

## Discussion

For static creatures, physical protection is limited against the enemies, as such, chemical compound becomes the second line of species defense, especially for those living in complex environments. Further, the last line of defense, genomic defense, play key roles in maintain the genetic stabilization of genome. The *Ganoderma* genus comprises a large group of saprobiotic mushroom-forming fungi that mostly live in subtropical/tropical regions, and many have economic value for applications in healthcare. Although the medicinal value of *Ganoderma spp.* has been extensively reported, their chemical and genomic defense machinery has never been elucidated.

Here, we report the genome sequence of *G. sinense*, one of the most valuable medicinal fungi. Together with the other two sequenced *Ganoderma* species, *G. lucidum* and *G. sp*, the genome sequence of this fungus provided further insight into the molecular basis and evolution of defense mechanisms in Basidiomycetes, particularly the genetic and epigenetic factors underlying the environmental induction of the defense systems and the production of secondary metabolites.

In this study, the combination of optical mapping and NGS technology is a powerful way to construct a chromosome-level genome sequence map. The *G. sinense* genome sequence was constructed into twelve chromosomal pseudomolecules based on the optical map scaffolds, which is one less than in *G. lucidum*. Additionally, the comparison of the genome sequences indicated a number of translocations and duplications among the three *Ganoderma* fungal species and also revealed many non-homologous genes, suggesting rapid divergent evolution following speciation. A considerable number of active TEs were also discovered in *G. sinense,* suggesting the association between specialization and rapid genome evolution^44^. Also, it suggests that the genome defense processes in this fungus may not be as strong as in the model species *N. crassa.*

Whole-genome bisulfite sequencing provided an overview of DNA methylation in *G. sinense* for the first time. Although DNA methylation was only noted on 1.8% of the cytosines in the genome, most of the methylated cytosines were distributed in repetitive regions. Accordingly, most cytosine methylation and RIP mutations were located in the same or adjacent regions (see Fig. 3C), suggesting the potential coordination of these mechanisms in TE silencing in *G. sinense*. Additionally, small double-stranded RNAs potentially generated from the transcription of both complimentary DNA strands in the subtelomeric regions were also identified, which may lead to RNA-mediated silencing of TE activity or telomeric heterochromatin.

As a fungi-specific genome defense system, RIP has been well studied in the model fungus *N. crassa*; however, this process remains largely unstudied in Basidiomycetes. We systemically investigated the RIP process in *G. sinense* and found that RIP mutations occurred most frequently on cytosine residues in CpG dinucleotides, compared to the CpA dinucleotides in *N. crassa* and most other fungi investigated to date^45^. CpG dinucleotides are also the most common targets of DNA methylation. Additionally, the key protein required for RIP, RID (*RIP defective*) in *N. crassa* (see Fig. 3), exhibits methyltransferase activity^46^. This raises the possibility that RID evolved from DNA methyltransferases and, therefore, that ancient RIDs may have preferentially recognized CpG dinucleotides. CpA is the second-most preferred dinucleotide for RIP in *G. sinense*. Conversion of CpG to TpG via RIP can introduce the stop codon TGA, while conversion of CpA to TpA can produce the stop codons, TAG and TAA. The introduction of stop codons can truncate the proteins essential for TE movement; therefore, RIP represents an efficient TE inactivation mechanism^47^.

Small RNAs can functionally produce the post-transcriptional gene silencing, some of which were found with genome defense related in many organisms^11^,^48^,^49^. In *G. sinense*, milRNAs and disiRNAs were first identified. Although there is no experimental evidence to be related to small RNA-mediated genome defense in *G. sinense*, the small RNAs derived from the TE enrichment regions may be involved in silencing or repressing transposable element activity for genome defense or for telomeric heterochromatin maintenance^50^,^51^,^52^.

Genome sequencing revealed that *G. sinense* encodes a large set of enzymes involved in the biosynthesis of secondary metabolites. Many of these genes appear to have originated from gene duplication, resulting in the potential for accelerated evolution via three fates: neofunctionalization, subfunctionalization and increased gene-dosage advantage^53^. The diversification of the cytochrome P450 (CYP) gene families in plants and higher fungi has led to the emergence of various biochemical pathways that produce secondary metabolites such as phenylpropanoids, alkaloids and terpenoids. Therefore, CYPs contribute to the chemical defense mechanisms of the organism^54^. Abundant CYPs have been identified in the Polyrales order^2^, which includes *G. sinense* and other traditional medicinal fungi, such as *G. lucidum*, *T. versicolor* and *W. cocos*. Thus, these species may have undergone expansion of CYPs for the enhanced biosynthesis of diverse secondary metabolites. In *G. sinense*, evidence for the tandem and fragmental duplication of CYPs provided some explanation for the gene expansion. The resulting diversification of CYPs during evolution could explain the divergence of triterpenoids between *G. sinense* and *G. lucidum*^1^,^32^,^55^.

More than thirty gene clusters were bioinformatically predicted in the *G. sinense* genome, and these gene clusters are expected to produce non-ribosomal peptide (NRPs), polyketide (PKs) or terpenoids. However, with the exception of triterpenoids, few NRPs, PKs or other types of terpenoids have been isolated and identified from this fungus. This suggests that most of these gene clusters are tightly controlled and silenced under general culturing conditions, resulting in the substantial underestimation of versatile secondary metabolite production. One way to maintain such tight control is through reversible gene silencing^17^,^56^; therefore, DNA methylation and small RNA-mediated gene silencing may play critical roles in genome defense and in the regulation of secondary metabolism. Considering that many secondary metabolites may play important roles in chemical defense, the co-cultivation of *G. sinense* with one or more microorganisms from the same ecosystem may be a promising way to activate specific gene clusters. This strategy has been demonstrated in *Aspergillus nidulans*^57^.

In conclusion, the analyses of the whole-genome sequence, DNA methylation and small RNA sequences suggest that *G. sinense* possesses the genetic and epigenetic hallmarks of genome defense systems, as well as chemical defense systems. Our data also provide new insights into the fundamental principles of secondary metabolite production in this and other important medicinal species, with significant implications for the pharmaceutical industry.

## Methods

### Strain and Culture Condition

*Ganoderma sinense* strain CGMCC5.0069 was sourced from the China General Microbiological Culture Collection Center (Beijing, China, http://www.cgmcc.net/). The haploid strain ZZ0214-1 was derived from CGMCC5.0069 using the protoplast monokaryogenesis method. In order to confirm the strain status, calcofluour white staining, DAPI staining and single nucleotide polymorphism (SNP) analysis were used as previously described 1. Liquid cultures were shaken in potato dextrose medium at 50 rpm for 6 days. The primordia and fruiting bodies of strain CGMCC5.0069 were cultivated on *Quercusvariabilis* Blume logs at HuiTao Pharmaceutical Company (LuoTian, Hubei Province, China). All strains are available upon request.

### Construction of Optical Maps

Protoplasts of the monokaryotic strain ZZ0214-1 were collected by centrifugation at 1,000 g for 10 min and were then diluted to a final concentration of 2 × 10^9^ cells mL^−1^. Gel inserts were prepared as previously described^19^ and stored in NDSK buffer (0.5 M EDTA, 1% (v/v) N-lauroylsarcosine, 1 mg/ml proteinase K). Genomic DNA was released by melting DNA gel inserts at 70 °C for 7 min, and then digesting with β-agarase (NEB, USA) at 42 °C for 2 h. Mounted DNA molecules were digested by the restriction endonuclease SpeI in NEB Buffer 2 (50 mM NaCl, 10 mM Tris-HCl, 10 mM MgCl_2_, 1 mM dithiothreitol, pH 7.9; New England Biolabs, USA) without BSA, and Triton X-100 was added to a final concentration of 0.02%. Digested DNA molecules were then stained with 12 μL of 0.2 μM YOYO-1 solution (5% YOYO-1 in TE containing 20% β-mercaptoethanol; Eugene, USA). Fully automated imaging workstations were used to generate single-molecule optical data sets for map assembly.

### Genome Sequencing and Assembly

Genomic DNA of *G. sinense* was sequenced using the Roche 454 GS FLX (Roche, USA) and Illumina HiSeq 2000 (Illumina, USA) NGS platforms. The sequencing process followed the manufacturer’s recommendations. Sequencing reads from Roche 454 were used to construct the primary assembly using CABOG^58^ and then scaffolded with Illumina paired-end reads using SSPACE version 1.1. The assembly was error checked and manually corrected to build the finished scaffolds. The final scaffolds were constructed to build the chromosome-wide pseudomolecules by optical mapping.

### Transcriptome Sequencing and Analysis

Gene expression analysis was performed on material obtained from the three developmental stages of *G. sinense*, including the mycelia, primordia, and fruiting bodies. Frozen samples were ground to powder in liquid nitrogen. Total RNA of each sample was extracted using an RNeasy Plant Mini Kit (Qiagen, Germany) according to manufacturer’s instructions but with 10% volume β–mercaptoethanol added to Buffer RLC before use. Genomic DNA was eliminated using DNase I (NEB, USA). RNA integrity and quality were checked using the RNA 6000 Nano II kit on a Bioanalyzer 2100 (Agilent, USA). RNA-Seq was performed as recommended by the manufacturer (Illumina, USA). And the samples were sequenced using an Illumina HiSeq 2000.

RNA-Seq data from the stages of mycelia, primordia and fruiting bodies were assembled to reconstruct the transcripts using Trinity. Transcript abundance was estimated as normalized fragments per kb of transcript per million mapped reads (FPKM) using RSEM. Differential expression analysis was executed using edgeR.

### Gene Annotation

Gene prediction was executed using the PASA software package^59^. Four software were used for *ab initio* gene finding, including Augustus, GeneMark, Fgenesh and SNAP. Additionally, the evidence-based method was used to weight and calibrate gene structure. The assembled transcripts and proteins were aligned to the genome assembly using exonerate:est2genome and exonerate:protein2genome, respectively. The predicted genes were functionally annotated by blasting the protein databases, including NCBI non-redundant, KEGG and Uniprot/Swissprot databases. Genes were also annotated using InterProScan, and then classified according to Gene Ontology terms.

### Genome Comparison and Phylogenetic Analysis

Syntenic genes between *G. sinense*, *G. lucidum* and *G. sp* were identified using MCscan (version 0.8). Orthologous groups from 41 fungal genomes were constructed using OrthoMCL version 2.0 (http://www.orthomcl.org). The BLASTP cutoff was set as E-value <1e-25. According to orthology analysis, 59 single copy proteins were selected and used for phylogenomic analysis. Individual alignments of the 59 proteins were generated by MUSCLE (version 3.6) from 25 species (see below) related to *G. sinense* and previously studied in genome defense, DNA methylation and RNA silencing. Using concatenated amino acid alignments, a maximum likelihood phylogenetic tree was subsequently produced by the program RAxML (version 7.2.8) with the PROTGAMMAWAG model and 100 bootstrap partitions. The divergence time between these fungi was estimated with penalized likelihood (PL) method using r8s by calibrating against three different clades in the Dikarya: Ascomycota (600–452 Ma), Boletales (130–50 Ma) and Agaricales (140–92 Ma)^20^.

Abbreviations of related 25 species: AspNi, *Aspergillus niger*; BatDe, *Batrachochytrium dendrobatidis*; ConPu, *Coniophora puteana*; CopCi, *Coprinopsis cinerea*; DicSq, *Dichomitus squalens*; FomMe, *Fomitiporia mediterranea*; FomPi, *Fomitopsis pinicola*; GanLu, *Ganoderma lucidum*; GanSi, *Ganoderma sinense*; GanSp, *Ganoderma sp.*; LacBi, *Laccaria bicolor*; MelLa, *Melampsora laricis-populina*; NeuCr, *Neurospora crassa*; PhaCh, *Phanerochaete chrysosporium*; PosPl, *Postia placenta*; PucGr, *Puccinia graminis f. sp. tritici*; RhoGr, *Rhodotorula graminis*; SacCe, *Saccharomyces cerevisiae*; SchCo, *Schizophyllum commune*; SchPo, *Schizosaccharomyces pombe*; SerLa, *Serpula lacrymans*; TraVe, *Trametes versicolor*; TreMe, *Tremella mesenterica*; UstMa, *Ustilago maydis*; WolCo, *Wolfiporia cocos*.

### Bisulfite Sequencing and Analysis of DNA Methylation

The Illumina bisulfite sequencing workflow was used to get the whole genome methylation profiles of *G. sinense* mycelia. Briefly, DNA was sheared to fragments of 100–500 bp using a Covaris. Fragments were polished using T4 DNA polymerase and Klenow enzyme. After end-repair, 3’ends were adenylated. Commercial adaptors were ligated to adenylated fragments and then purified. The purified products were treated twice with sodium bisulfite using the EpiTect Bisulfite Kit to convert unmethylated Cs to Us. The treated DNA was amplified by 18 cycles of PCR using high fidelity polymerase. Size-selection was conducted after amplification, bands around 300 bp were used for sequencing with the Illumina HiSeq 2000 platform.

DNA methylation analysis was performed using Bismark^60^, which maps bisulfite treated sequencing reads to the genome. Methylation level for each gene was estimated by counting the number of methylation sites per kilobase pairs.

### Small RNA Sequencing

Strand-specific small RNA sequencing was performed according to the Illumina TruSeq small RNA sequencing protocol. One microgram of total RNA from mycelia or fruiting bodies was used as input. After 3’ and 5’ adaptor ligation using T4 RNA ligase, reverse transcription and amplification were conducted using PCR as the manufacturer’s instruction. The amplification quality was detected using a high sensitivity DNA chip on a Bioanalyzer 2100. Amplified cDNA bands of around 150 bp were agarose gel-purified and concentrated by ethanol precipitation. Library validation was conducted on an Agilent 2100 Bioanalyzer using a high sensitivity DNA chip. Sequencing was performed on an Illumina HiSeq 2000 platform.

### Identification of Small RNAs

Small RNAs between 18–30 nt were identified in our study. The loci of milRNAs were determined by the following criteria^24^: 1) Small RNAs were significantly derived from one DNA strand (at least 10 fold more than the other DNA strands); 2) Small RNAs were highly enriched at the loci with a density over 270 reads/kb; 3) Small RNAs were located on the stem region of a hairpin structure with flanking sequences using secondary structures predicted by RNAfold (http://rna.tbi.univie.ac.at/cgi-bin/RNAfold.cgi); 4) Sequences with a minimum folding energy less than −20 kcal/mol and a minimal folding free energy index (MFEI) greater than 0.8 was considered as milRNA precursors.

disiRNA loci were identified by screening the whole genome. Two criteria were used to predict disiRNAs^24^: 1) Similar abundance from both DNA strands; 2) The density of small RNAs was significantly higher than the background (at least 60 reads/kb).

### Transposable Elements and RIP Analysis

Transposable elements were identified and annotated by the REPET package (http://urgi.versailles.inra.fr/Tools/REPET), which contains TEdenovo and TEannot pipelines. TEs with a minimum length of 400 bp and greater than 80% sequence identity were used for expression analysis. Expression of TEs was evaluated by mapping RNA-Seq sequences to the genome using TopHat version 2.0.9. Expressed TEs were defined according to following criteria: 1) RNA-Seq reads mapped to the genome reference sequence allowing two mismatches; 2) Paired reads that spanned over estimated inserts or mapped to different scaffolds were discarded; 3) Reads with multiple hits were filtered. Then, the remaining reads were used to determine TE expression.

TEs with a minimum length of 400 bp and greater than 80% sequence identity were also used for RIP analysis. To detect RIP sites, an alignment-based method was used to locate the variant sites according to the characteristics of C to T variation. Pre-RIP sites, which represent the state prior to RIP mutation, were also determined by sequence alignment. RIP indices were calculated using RIPCAL.

## Additional Information

**Accession Numbers** The assembled *G. sinense* genome and gene resources were submitted to GenBank (Bioproject: SRP026627). Raw reads of genome, transcriptome, DNA methylation and small RNA sequencing were deposited under SRA with accession numbers: SRR927381, SRR927409 - SRR927414 for genome sequencing; SRR929381, SRR933611, SRR929382, SRR929435 for transcriptome sequencing; SRR929357 for bisulfite sequencing; and SRR929280 and SRR1562261 for small RNA sequencing.

**How to cite this article**: Zhu, Y. *et al.* Chromosome-level genome map provides insights into diverse defense mechanisms in the medicinal fungus *Ganoderma sinense*. *Sci. Rep.* **5**, 11087; doi: 10.1038/srep11087 (2015).

## Supplementary Material

Supplementary Information

## Figures and Tables

**Table 1:** **Genome characteristics of** ***G. sinense***.

Number of chromosomes	12
Number of scaffolds	69
Length of genome assembly (Mb)	48.96
Scaffold L50 (Mb)	2.26
Scaffold N50	8
GC content (%)	55.59
Number of protein-coding genes	15,688
Average gene length (bp)	1,663
GC content of protein-coding genes (%)	57.93
Average number of exons per gene	5
Average exon size (bp)	245
Average coding sequence size (bp)	1,308
Average intron size (bp)	82
Average size of intergenic regions (bp)	1,451
Number of tRNA (pseudogenes)	202 (9)

**Table 2:** **Expression of transposable elements in** ***G. sinense***.

			**CLASSI retrotransposons (%)**				**CLASSII transposons (%)**			
**TE**^**a**^	**Exp**^**b**^	**Num**^**c**^	**LINE**	**LTR**	**DIRS**	**PLE**	**TIR**	***helitron***	**MITE**	**?**^**d**^
Total	Yes	425	3.13	4.65	0.04	0.00	6.26	0.04	1.12	0.04
	No	2,067	2.69	59.71	0.48	0.48	10.91	1.36	3.21	0.00
RIP	Yes	242	0.60	3.01	0.00	0.00	4.49	0.04	0.84	0.00
	No	1,514	1.52	45.06	0.16	0.48	6.54	0.60	3.05	0.00
Non-RIP	Yes	183	2.53	1.65	0.04	0.00	1.77	0.00	0.28	0.04
	No	553	1.16	14.65	0.32	0.00	4.37	0.76	0.16	0.00
5 mC	Yes	189	0.80	3.33	0.04	0.00	2.41	0.04	0.16	0.00
	No	1,930	2.45	58.95	0.48	0.48	8.35	1.36	1.44	0.00
Non-5 mC	Yes	236	2.33	1.32	0.00	0.00	3.85	0.00	0.96	0.04
	No	137	0.24	0.76	0.00	0.00	2.57	0.00	1.77	0.00
RIP & 5 mC	Yes	144	0.32	2.85	0.00	0.00	1.89	0.04	0.08	0.00
	No	1,413	1.44	44.82	0.16	0.48	4.49	0.60	1.44	0.00
Non-RIP & non-5 mC	Yes	138	2.05	1.16	0.00	0.00	1.24	0.00	0.20	0.04
	No	36	0.16	0.52	0.00	0.00	0.52	0.00	0.16	0.00

The percentage of expressed or non-expressed CLASSI and CLASSII TEs showing RIP or methylation modifications were calculated relative to the total number of TEs. Only TEs having high identity with other TEs (>80%) are represented in this table. TE ^a^ delineates the TEs that were subject to RIP mutations or cytosine methylation. Exp^b^ refers to the status of expression; Num^c^ is the number of TEs. ?^d^ represents unclassified DNA transposons.

## References

[b1] Chen S. *et al.* Genome sequence of the model medicinal mushroom *Ganoderma lucidum*. Nat. Commun. 3, 913 (2012). 22735441 10.1038/ncomms1923 PMC3621433

[b2] Syed K., Nelson D. R., Riley R. & Yadav J. S. Genome-wide annotation and comparative genomics of cytochrome P450 monooxygenases (P450s) in the Polyporale species *Bjerkandera adusta*, *Ganoderma sp.* and *Phlebia brevispora*. Mycologia 105, 1445–1455 (2013). 23928414 10.3852/13-002

[b3] Rountree M. R. & Selker E. U. Genome Defense: The Neurospora Paradigm in *Epigenomics* (ed. Ferguson-Smith A. C. *et al.*) 321–341 10.1007/978-1-4020-9187-2_18 (Springer: Netherlands, , 2009).

[b4] Selker E. U., Cambareri E. B., Jensen B. C. & Haack K. R. Rearrangement of duplicated DNA in specialized cells of *Neurospora*. Cell 51, 741–752 (1987). 2960455 10.1016/0092-8674(87)90097-3

[b5] Galagan J. E. & Selker E. U. RIP: the evolutionary cost of genome defense. Trends in Genetics : TIG 20, 417–423 (2004). 15313550 10.1016/j.tig.2004.07.007

[b6] Galagan J. E. *et al.* The genome sequence of the filamentous fungus *Neurospora crassa*. Nature 422, 859–868 (2003). 12712197 10.1038/nature01554

[b7] Perkins D. D., Margolin B. S., Selker E. U. & Haedo S. D. Occurrence of repeat induced point mutation in long segmental duplications of *Neurospora*. Genetics 147, 125–136 (1997). 9286673 10.1093/genetics/147.1.125 PMC1208096

[b8] Selker E. U. Genome defense and DNA methylation in *Neurospora*. Cold Spring Harb. Symp. Quant. Biol. 69, 119–124 (2004). 16117640 10.1101/sqb.2004.69.119

[b9] Zemach A., McDaniel I. E., Silva P. & Zilberman D. Genome-wide evolutionary analysis of eukaryotic DNA methylation. Science 328, 916–919 (2010). 20395474 10.1126/science.1186366

[b10] Selker E. U. *et al.* The methylated component of the *Neurospora crassa* genome. Nature 422, 893–897 (2003). 12712205 10.1038/nature01564

[b11] Moazed D. Small RNAs in transcriptional gene silencing and genome defence. Nature 457, 413–420 (2009). 19158787 10.1038/nature07756 PMC3246369

[b12] Chang S. S., Zhang Z. & Liu Y. RNA interference pathways in fungi: mechanisms and functions. Annu. Rev. Microbiol. 66, 305–323 (2012). 22746336 10.1146/annurev-micro-092611-150138 PMC4617789

[b13] Dang Y., Yang Q., Xue Z. & Liu Y. RNA interference in fungi: pathways, functions, and applications. Eukaryot. cell 10, 1148–1155 (2011). 21724934 10.1128/EC.05109-11 PMC3187057

[b14] Mousa W. K. & Raizada M. N. The diversity of anti-microbial secondary metabolites produced by fungal endophytes: an interdisciplinary perspective. Front. Microbiol. 4, 65, (2013). 23543048 10.3389/fmicb.2013.00065 PMC3608919

[b15] Rohlfs M. & Churchill A. C. Fungal secondary metabolites as modulators of interactions with insects and other arthropods. Fungal Genet. Biol. 48,23–34 (2011). 20807586 10.1016/j.fgb.2010.08.008

[b16] Keller N. P., Turner G. & Bennett J. W. Fungal secondary metabolism - from biochemistry to genomics. Nat. Rev. Microbiol. 3, 937–947 (2005). 16322742 10.1038/nrmicro1286

[b17] Brakhage A. A. Regulation of fungal secondary metabolism. Nat. Rev. Microbiol. 11, 21–32, (2013). 23178386 10.1038/nrmicro2916

[b18] Zhou S. *et al.* Whole-genome shotgun optical mapping of *Rhodobacter sphaeroides* strain 2.4.1 and its use for whole-genome shotgun sequence assembly. Genome Res. 13, 2142–2151, (2003). 12952882 10.1101/gr.1128803 PMC403714

[b19] Zhou S. *et al.* A single molecule scaffold for the maize genome. PLoS Genet. 5, (2009). 10.1371/journal.pgen.1000711 PMC2774507 19936062

[b20] Floudas D. *et al.* The Paleozoic origin of enzymatic lignin decomposition reconstructed from 31 fungal genomes. Science 336, 1715–1719 (2012). 22745431 10.1126/science.1221748

[b21] Hood M. E., Katawczik M. & Giraud T. Repeat-induced point mutation and the population structure of transposable elements in *Microbotryum violaceum*. Genetics 170, 1081–1089, (2005). 15911572 10.1534/genetics.105.042564 PMC1451165

[b22] Watters M. K., Randall T. A., Margolin B. S., Selker E. U. & Stadler D. R. Action of repeat-induced point mutation on both strands of a duplex and on tandem duplications of various sizes in *Neurospora*. Genetics 153, 705–714 (1999). 10511550 10.1093/genetics/153.2.705 PMC1460768

[b23] Lewis Z. A. *et al.* Relics of repeat-induced point mutation direct heterochromatin formation in *Neurospora crassa*. Genome Res. 19, 427–437 (2009). 19092133 10.1101/gr.086231.108 PMC2661801

[b24] Lee H. C. *et al.* Diverse pathways generate microRNA-like RNAs and Dicer-independent small interfering RNAs in fungi. Mol. Cell 38, 803–814 (2010). 20417140 10.1016/j.molcel.2010.04.005 PMC2902691

[b25] Choi J. *et al.* funRNA: a fungi-centered genomics platform for genes encoding key components of RNAi. BMC Genomics 15, S14 (2014). 25522231 10.1186/1471-2164-15-S9-S14 PMC4290597

[b26] Boh B., Berovic M., Zhang J. & Zhi-Bin L. *Ganoderma lucidum* and its pharmaceutically active compounds. Biotechnol. Annu. Rev. 13, 265–301 (2007). 17875480 10.1016/S1387-2656(07)13010-6

[b27] Sanodiya B. S., Thakur G. S., Baghel R. K., Prasad G. B. & Bisen P. S. *Ganoderma lucidum*: a potent pharmacological macrofungus. Curr. Pharm. Biotechnol. 10, 717–742 (2009). 19939212 10.2174/138920109789978757

[b28] Zhao J. *et al.* Quality evaluation of *Ganoderma* through simultaneous determination of nine triterpenes and sterols using pressurized liquid extraction and high performance liquid chromatography. J. Sep. Sci. 29, 2609–2615 (2006). 17313101 10.1002/jssc.200600178

[b29] Da J. *et al.* Comparison of two officinal Chinese pharmacopoeia species of *Ganoderma* based on chemical research with multiple technologies and chemometrics analysis. J. Chromatogr. A 1222, 59–70 (2012). 22226558 10.1016/j.chroma.2011.12.017

[b30] Blin K. *et al.* antiSMASH 2.0–a versatile platform for genome mining of secondary metabolite producers. Nucleic. Acids Res. 41, W204–W212 (2013). 23737449 10.1093/nar/gkt449 PMC3692088

[b31] Khaldi N. *et al.* SMURF: Genomic mapping of fungal secondary metabolite clusters. Fungal Genet. Biol. 47, 736–741 (2010). 20554054 10.1016/j.fgb.2010.06.003 PMC2916752

[b32] Ide M., Ichinose H. & Wariishi H. Molecular identification and functional characterization of cytochrome P450 monooxygenases from the brown-rot basidiomycete *Postia placenta*. Arch. Microbiol. 194, 243–253 (2012). 21938516 10.1007/s00203-011-0753-2

[b33] Nelson D. R. Progress in tracing the evolutionary paths of cytochrome P450. Biochim. Biophys. Acta 1814, 14–18 (2011). 20736090 10.1016/j.bbapap.2010.08.008

[b34] Yin W. & Keller N. P. Transcriptional regulatory elements in fungal secondary metabolism. J. Microbiol. 49, 329–339 (2011). 21717315 10.1007/s12275-011-1009-1 PMC3714018

[b35] Dowzer C. E. & Kelly J. M. Cloning of the *creA* gene from *Aspergillus nidulans*: a gene involved in carbon catabolite repression. Curr. Genet. 15, 457–459 (1989). 2673558 10.1007/BF00376804

[b36] Dowzer C. E. & Kelly J. M. Analysis of the *creA* gene, a regulator of carbon catabolite repression in *Aspergillus nidulans*. Mol. Cell. Biol. 11, 5701–5709 (1991). 1922072 10.1128/mcb.11.11.5701-5709.1991 PMC361941

[b37] Tilburn J. *et al.* The *Aspergillus PacC* zinc finger transcription factor mediates regulation of both acid- and alkaline-expressed genes by ambient pH. EMBO J. 14, 779–790 (1995). 7882981 10.1002/j.1460-2075.1995.tb07056.x PMC398143

[b38] Christensen T., Hynes M. J. & Davis M. A. Role of the regulatory gene *areA* of *Aspergillus oryzae* in nitrogen metabolism. Appl. Environ. Microbiol. 64, 3232–3237 (1998). 9726865 10.1128/aem.64.9.3232-3237.1998 PMC106715

[b39] Calvo A. M. The VeA regulatory system and its role in morphological and chemical development in fungi. Fungal. Genet. Biol. 45, 1053–1061 (2008). 18457967 10.1016/j.fgb.2008.03.014

[b40] Bayram O. *et al.* VelB/VeA/LaeA complex coordinates light signal with fungal development and secondary metabolism. Science 320, 1504–1506 (2008). 18556559 10.1126/science.1155888

[b41] Yazaki K. Transporters of secondary metabolites. Curr. Opin. Plant. Biol. 8, 301–307 (2005). 15860427 10.1016/j.pbi.2005.03.011

[b42] Yazaki K. ABC transporters involved in the transport of plant secondary metabolites. FEBS letters 580, 1183–1191 (2006). 16364309 10.1016/j.febslet.2005.12.009

[b43] Kovalchuk A. & Driessen A. J. Phylogenetic analysis of fungal ABC transporters. BMC Genomics 11, 177 (2010). 20233411 10.1186/1471-2164-11-177 PMC2848647

[b44] Hu X. *et al.* Trajectory and genomic determinants of fungal-pathogen speciation and host adaptation. Proc. Natl. Acad. Sci. USA 111, 16796–16801 (2014). 25368161 10.1073/pnas.1412662111 PMC4250126

[b45] Clutterbuck A. J. Genomic evidence of repeat-induced point mutation (RIP) in filamentous ascomycetes. Fungal. Genet. Biol. 48, 306–326 (2011). 20854921 10.1016/j.fgb.2010.09.002

[b46] Freitag M., Williams R. L., Kothe G. O. & Selker E. U. A cytosine methyltransferase homologue is essential for repeat-induced point mutation in *Neurospora crassa*. Proc. Natl. Acad. Sci. USA 99, 8802–8807 (2002). 12072568 10.1073/pnas.132212899 PMC124379

[b47] Fudal I. *et al.* Repeat-induced point mutation (RIP) as an alternative mechanism of evolution toward virulence in *Leptosphaeria maculans*. Mol. Plant. Microbe. InteractI. 22, 932–941 (2009). 10.1094/MPMI-22-8-0932 19589069

[b48] Singh D. P. *et al.* Genome-defence small RNAs exapted for epigenetic mating-type inheritance. Nature 509, 447–452 (2014). 24805235 10.1038/nature13318

[b49] Creasey K. M. *et al.* miRNAs trigger widespread epigenetically activated siRNAs from transposons in *Arabidopsis*. Nature 508, 411–415 (2014). 24670663 10.1038/nature13069 PMC4074602

[b50] Grewal S. I. & Jia S. Heterochromatin revisited. Nat. Rev. Genet. 8, 35–46 (2007). 17173056 10.1038/nrg2008

[b51] Moazed D. *et al.* Studies on the mechanism of RNAi-dependent heterochromatin assembly. Cold Spring Harb. Symp. Quant. Biol. 71, 461–471 (2006). 17381328 10.1101/sqb.2006.71.044

[b52] Cao F. *et al.* Dicer independent small RNAs associate with telomeric heterochromatin. RNA 15, 1274–1281 (2009). 19460867 10.1261/rna.1423309 PMC2704082

[b53] Pegueroles C., Laurie S. & Alba M. M. Accelerated evolution after gene duplication: a time-dependent process affecting just one copy. Mol. Biol. Evol. 30, 1830–1842 (2013). 23625888 10.1093/molbev/mst083

[b54] Mizutani M. & Ohta D. Diversification of P450 genes during land plant evolution. Annu. Rev. Plant. Biol. 61, 291–315 (2010). 20192745 10.1146/annurev-arplant-042809-112305

[b55] Cresnar B. & Petric S. Cytochrome P450 enzymes in the fungal kingdom. Biochim. Biophys. Acta 1814, 29–35 (2011). 20619366 10.1016/j.bbapap.2010.06.020

[b56] Shwab E. K. *et al.* Histone deacetylase activity regulates chemical diversity in *Aspergillus*. Eukaryot. Cell 6, 1656–1664 (2007). 17616629 10.1128/EC.00186-07 PMC2043372

[b57] Schroeckh V. *et al.* Intimate bacterial-fungal interaction triggers biosynthesis of archetypal polyketides in *Aspergillus nidulans*. Proc Natl Acad Sci USA 106, 14558–14563 (2009). 19666480 10.1073/pnas.0901870106 PMC2732885

[b58] Miller J. R. *et al.* Aggressive assembly of pyrosequencing reads with mates. Bioinformatics 24, 2818–2824 (2008). 18952627 10.1093/bioinformatics/btn548 PMC2639302

[b59] Haas B. J. *et al.* Improving the Arabidopsis genome annotation using maximal transcript alignment assemblies. Nucleic. Acids Res. 31, 5654–5666 (2003). 14500829 10.1093/nar/gkg770 PMC206470

[b60] Krueger F., & Andrews S. R. Bismark: a flexible aligner and methylation caller for Bisulfite-Seq applications. Bioinformatics 27: 1571–1572 (2011). 21493656 10.1093/bioinformatics/btr167 PMC3102221

