# Supplementary material for: Chromosome-level genome map provides insights into diverse defense mechanisms in the medicinal fungus Ganoderma sinense
Source: Sci Rep. 2015 Jun 5;5:11087. doi: 10.1038/srep11087 (PMC4457147; doi:10.1038/srep11087)
Supplement: Supplementary Information [file srep11087-s1.pdf]

Chromosome-level genome map provides insights into diverse defense mechanisms in the medicinal fungus *Ganoderma sinense*

Yingjie Zhu[1,2 §], Jiang Xu[1,2 §], Chao Sun[2,*], Shiguo Zhou[3], Haibin Xu[1], David R. Nelson[4], Jun Qian[2], Jingyuan Song[2], Hongmei Luo[2], Li Xiang[2], Ying Li[2], Zhichao Xu[2], Aijia Ji[2], Lizhi Wang[2], Shanfa Lu[2], Alice Hayward[5], Wei Sun[1], Xiwen Li[1], David C. Schwartz[3], Yitao Wang[6], Shilin Chen[1,2,*]

Supplementary figures

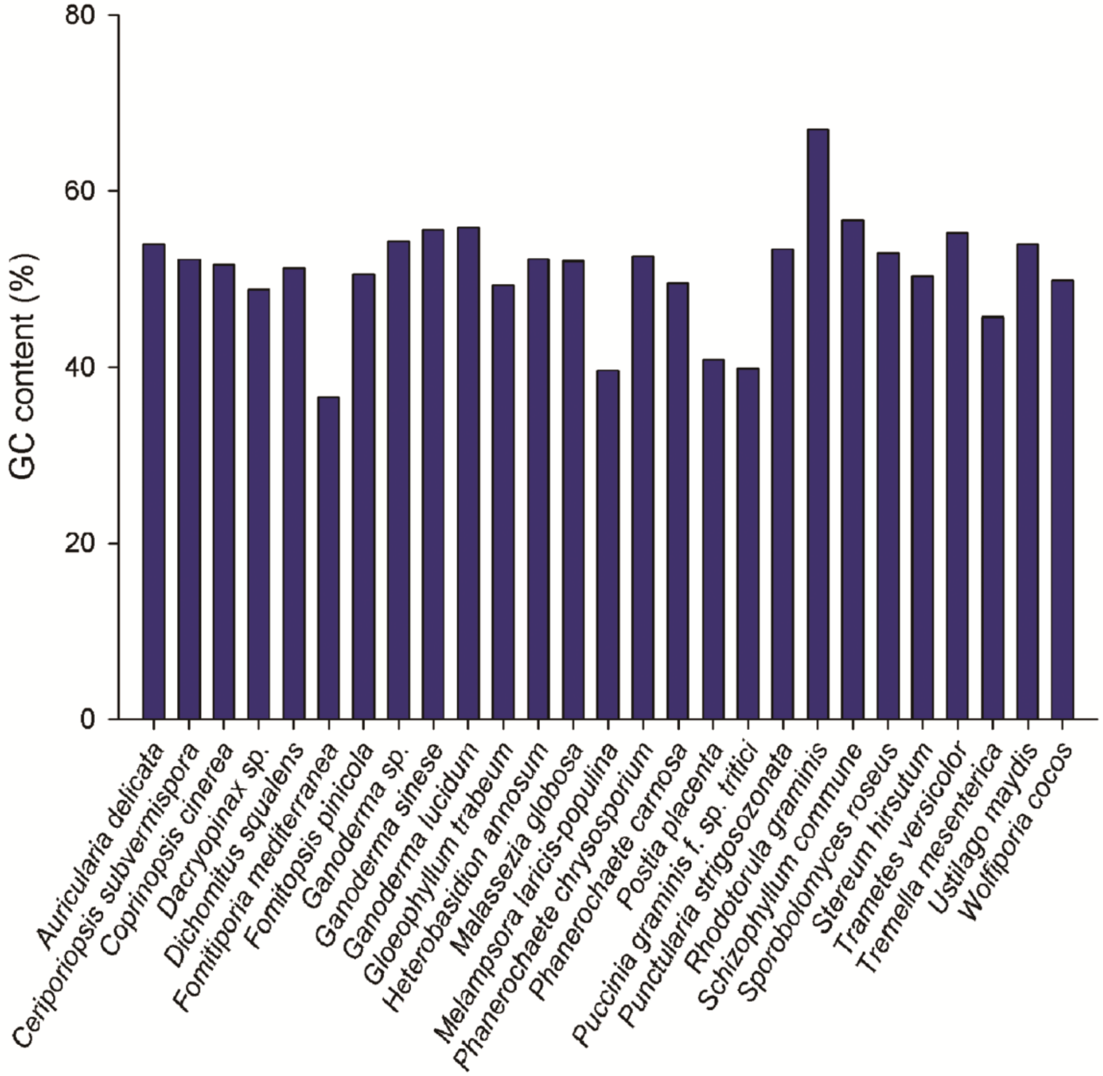

Supplementary Figure S1. The average of GC content of Basidiomycota genomes.

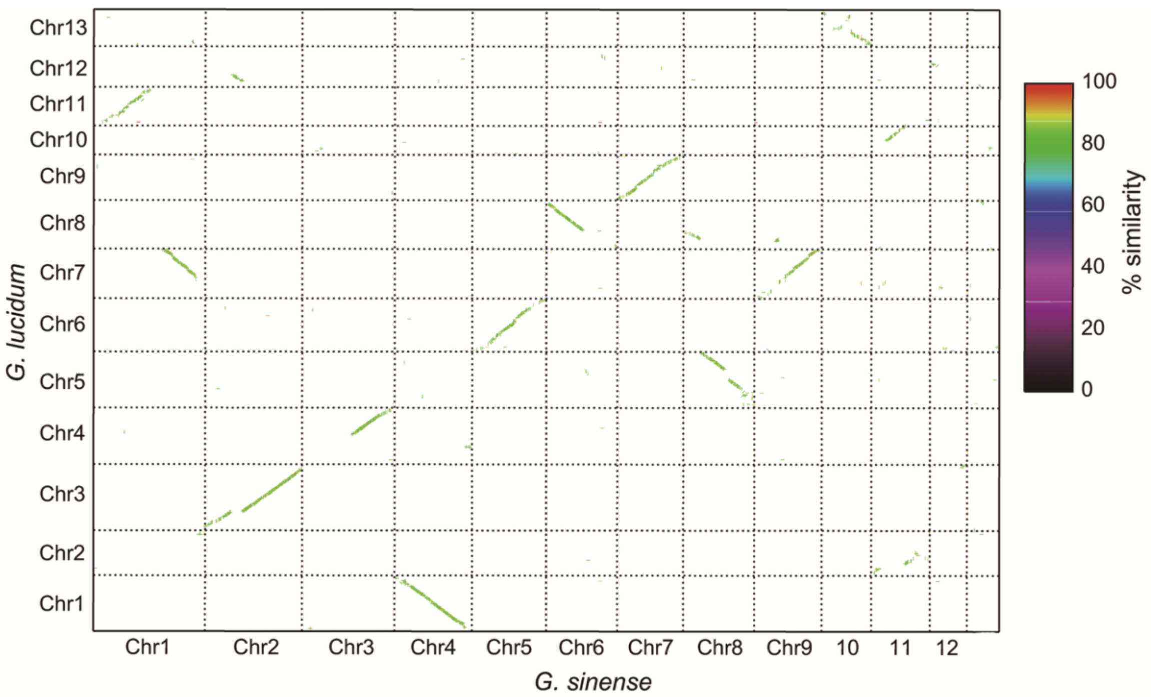

Supplementary Figure S2. Dot plot of the *G. sinense* and *G. lucidum* genomes. Nucleotide comparison on the chromosome level was executed by NUCmer in the MUMmer software package. Green lines show blocks of ~80% similarity between *G. sinense* and *G. lucidum*.

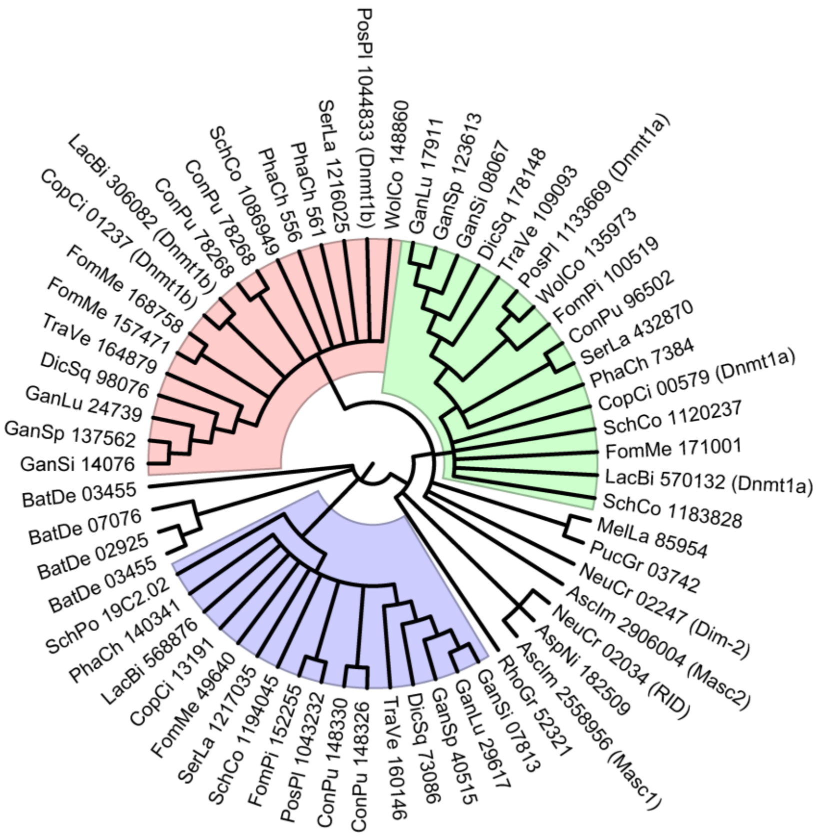

Supplementary Figure S3. Unrooted phylogram of DNA methyltransferase homologs in 23 fungi. The maximum parsimony method was used to construct a phylogenetic tree using the PAUP4b10 software with 1000 bootstraps. The Dnmt1a, Dnmt1b and Dnmt2 subfamilies are shaded in green, red and purple, respectively.

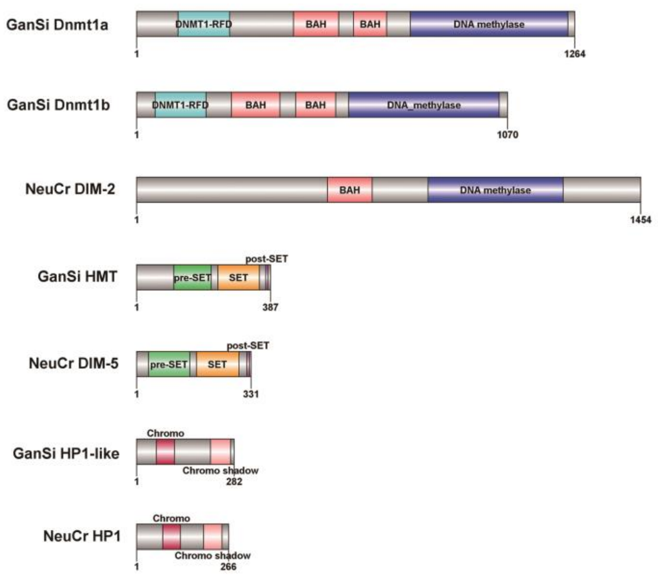

Supplementary Figure S4. Domain structures of DMTase (DNA methyltransferase), HMT (Histone methyltransferase) and HP1 (Heterochromatin Protein 1) for *G. sinense* and *N. crassa*. Domains were predicted using PfamScan (version 27.0) with a cutoff of e_seq < 0.01 and e_dom < 0.03. Each color represents a specific domain as shown.

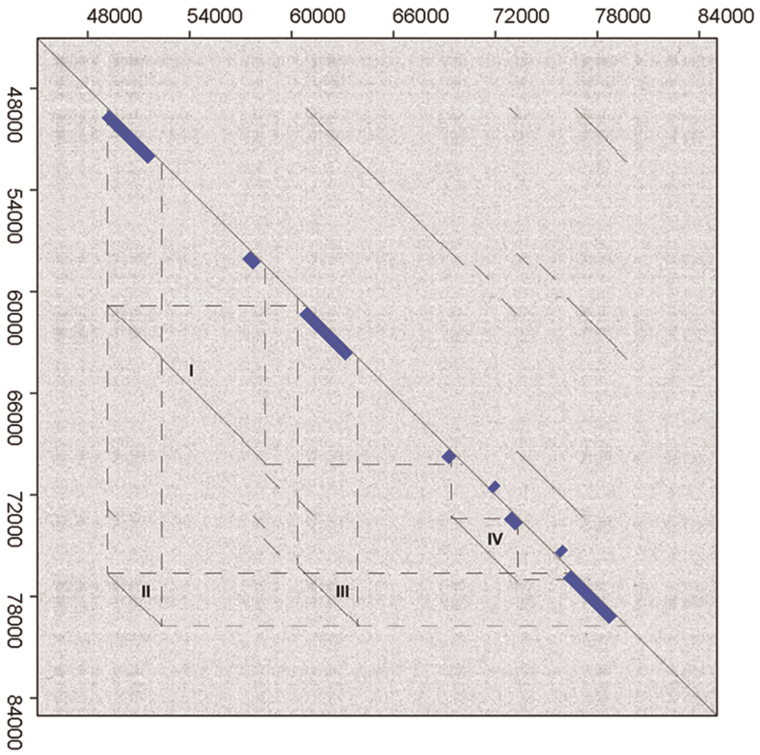

Supplementary Figure S5. Fragmental duplications occurred in a methylated CYP gene cluster. A fragment within scaffold 44, which contained CYP gene clusters, was compared with itself to detect internal repeats using the dot-plot method. The diagonal line represents the 100% self-match. The duplicated fragments are shown as the other line segments. The genes on the positive strand are shown as blue rectangles above the diagonal; genes on the negative strand are shown under the diagonal. Four major duplication events are marked with roman numerals.

Supplementary Tables

Supplementary Table S1. Sequencing statistics in this project.

Library	Library ID	Sequencing Platform	Insert Size (kb)	No. of Reads	Average length (bp)
Genome sequencing	1	Roche FLX Titanium	0	1,695,406	433
	2	Roche FLX Titanium	3	1,579,667	312
	3	Roche FLX Titanium	8	335,495	351
	4	Roche FLX Titanium	20	453,560	400
	5	Illumina HiSeq 2000	0.3	50,239,835	100*2
	6	Illumina HiSeq 2000	5	55,465,866	100*2
Transcriptome sequencing	7	Illumina HiSeq 2000	0.3	28,262,342	100*2
	8	Illumina HiSeq 2000	0.3	22,839,001	100*2
	9	Illumina HiSeq 2000	0.3	15,948,084	100*2
Bisulfite sequencing	10	Illumina HiSeq 2000	0.3	24,789,969	100*2
Small RNA sequencing	11	Illumina HiSeq 2000	0	12,912,296	32
	12	Illumina HiSeq 2000	0	11,521,487	19

Supplementary Table S2. Transposable elements in *G. sinense*, *G. lucidum* and *G. sp*.

Elements	*G. sinense*		*G. lucidum*		*G. sp.*	
	Total	Size (bp)	Total	Size (bp)	Total	Size (bp)

	number		number		number	
**CLASSI Retrotransposons**	23,659	6,535,872	18,827	6,122,828	10,296	3,312,677
**DIRS**	32	17,455	0	0	43	18,149
**LINE**	5,287	1,499,495	238	96,028	222	45,511
**LTR**	18,310	5,004,801	18,491	6,001,510	10,001	3,240,920
Gypsy	10,014	1,858,453	10,792	2,309,873	6,535	1,428,434
Copia	3,652	739,048	3,551	801,595	1,074	255,947
**PLE**	21	13,107	0	0	24	7,489
**Others**	9	1,014	98	25,290	6	608
**CLASSII Transposons**	2,479	1,076,397	2,686	984,605	1,069	470,858
helitron	364	119,721	93	36,539	96	25,149
**MAVERICK**	1	562	0	0	1	640
**MITE**	319	101,341	92	30,670	43	16,477
**TIR**	1,779	851,219	2,500	916,458	922	425,788
**POLINTON**	0	0	1	938	0	0
**Others**	16	3,554	0	0	7	2,804
**Unclassified**	1,568	765,594	1,400,183	0	566	361,775

Supplementary Table S3. Data source for genome comparison and phylogenetic analysis.

**Species**	**Abbreviation**	**Source**	**Website**
Ascobolus immersus	AscIm	NCBI	http://www.ncbi.nlm.nih.gov/
Aspergillus niger	AspNi	JGI	http://www.jgi.doe.gov/
Batrachochytrium dendrobatidis	BatDe	BROAD	http://broadinstitute.org/
Coniophora puteana	ConPu	JGI	http://www.jgi.doe.gov/
Coprinopsis cinerea	CopCi	BROAD	http://broadinstitute.org/
Dichomitus squalens	DicSq	JGI	http://www.jgi.doe.gov/
Fomitiporia mediterranea	FomMe	JGI	http://www.jgi.doe.gov/
Fomitopsis pinicola	FomPi	JGI	http://www.jgi.doe.gov/
Ganoderma lucidum	GanLu	NCBI	http://www.ncbi.nlm.nih.gov/
Ganoderma sinense	GanSi	NCBI	http://www.ncbi.nlm.nih.gov/
Ganoderma sp.	GanSp	JGI	http://www.jgi.doe.gov/
Laccaria bicolor	LacBi	JGI	http://www.jgi.doe.gov/
Melampsora laricis-populina	MelLa	JGI	http://www.jgi.doe.gov/
Neurospora crassa	NeuCr	BROAD	http://broadinstitute.org/
Phanerochaete chrysosporium	PhaCh	JGI	http://www.jgi.doe.gov/
Postia placenta	PosPl	JGI	http://www.jgi.doe.gov/
Puccinia graminis f. sp. tritici	PucGr	BROAD	http://broadinstitute.org/

Rhodotorula graminis	RhoGr	JGI	http://www.jgi.doe.gov/
Saccharomyces cerevisiae	SacCe	JGI	http://www.jgi.doe.gov/
Schizophyllum commune	SchCo	JGI	http://www.jgi.doe.gov/
Schizosaccharomyces pombe	SchPo	BROAD	http://broadinstitute.org/
Serpula lacrymans	SerLa	JGI	http://www.jgi.doe.gov/
Trametes versicolor	TraVe	JGI	http://www.jgi.doe.gov/
Tremella mesenterica	TreMe	JGI	http://www.jgi.doe.gov/
Ustilago maydis	UstMa	BROAD	http://broadinstitute.org/
Wolfiporia cocos	WolCo	JGI	http://www.jgi.doe.gov/

Supplementary Table S4. Comparison of DNA methyltransferases among 25 fungi.

These DNA methyltransferases were identified based on the analysis of sequence similarity, Pfam domain and phylogenetic tree.

**Species**	**DNMT1**	**DNMT2**	**DNMT3**	**DIM-2**	**Masc1**	**Masc2**	**RID**
Aspergillus niger	0	0	0	0	1	0	0
Batrachochytrium dendrobatidis	0	4	0	0	0	0	0
Coniophora puteana	3	2	0	0	0	0	0
Coprinopsis cinerea	2	1	0	0	0	0	0
Dichomitus squalens	2	1	0	0	0	0	0

Fomitiporia mediterranea	3	1	0	0	0	0	0
Fomitopsis pinicola	1	1	0	0	0	0	0
Ganoderma lucidum	2	1	0	0	0	0	0
Ganoderma sinense	2	1	0	0	0	0	0
Ganoderma sp.	2	1	0	0	0	0	0
Laccaria bicolor	2	1	0	0	0	0	0
Melampsora laricis-populina	0	0	0	0	0	1	0
Neurospora crassa	0	0	0	1	0	0	1
Phanerochaete chrysosporium	3	1	0	0	0	0	0
Postia placenta	2	1	0	0	0	0	0
Puccinia graminis f. sp. tritici	1	0	0	0	0	0	0
Rhodotorula graminis	1	0	0	0	0	0	0
Saccharomyces cerevisiae	0	0	0	0	0	0	0
Schizophyllum commune	3	1	0	0	0	0	0
Schizosaccharomyces pombe	0	1	0	0	0	0	0
Serpula lacrymans	2	1	0	0	0	0	0
Trametes versicolor	2	1	0	0	0	0	0

Tremella mesenterica	0	0	0	0	0	0	0
Ustilago maydis	0	0	0	0	0	0	0
Wolfiporia cocos	2	0	0	0	0	0	0

Supplementary Table S5. Genes related to DNA methylation and histone methylation.

Gene family/name	Gene ID	EC number	Species (hits)	Accession	Similarity
DNMT/*Dnmt1a*	GS08067	EC:2.1.1.37	*Postia placenta*	XP_002474649	46%
DNMT/*Dnmt1b*	GS14076	EC:2.1.1.37	*Postia placenta*	XP_002472324	36%
DNMT/*Dnmt2*	GS07813	EC:2.1.1.37	*Coprinopsis cinerea*	XP_001828513	53%
Chromobox protein /*HP1*	GS13057		*Coprinopsis cinerea*	XP_001831718	41%
HMT/*HMT*	GS01733	EC:2.1.1.43	*Schizosaccharomyces pombe*	NP_595186	36%
DNA damage-binding protein/*DDB1*	GS15309		*Coprinopsis cinerea*	XP_001835883	37%
Cullin/*CUL4*	GS07008		*Coprinopsis cinerea*	XP_001828306	42%

Supplementary Table S6. Domain organizations of DNA methyltransferases.

Species	Protein ID	Putative	BAH*	DNA	DNMT1-

		family		methylase*	RFD*
Ascobolus immersus	2558956	Masc1	1	2	
Ascobolus immersus	2906004	Masc2	2	1	1
Aspergillus niger	182509	Masc1		2	
Batrachochytrium dendrobatidis	1375	Dnmt2		1	
Batrachochytrium dendrobatidis	2925	Dnmt2		1	
Batrachochytrium dendrobatidis	3455	Dnmt2		1	
Batrachochytrium dendrobatidis	7076	Dnmt2		1	
Coniophora puteana	96502	Dnmt1a	1	1	1
Coniophora puteana	162212	Dnmt1b		2	1
Coniophora puteana	78268	Dnmt1b	1	2	1
Coniophora puteana	148326	Dnmt2a		1	
Coniophora puteana	148330	Dnmt2b		1	
Coprinopsis cinerea	579	Dnmt1a	1	1	1
Coprinopsis cinerea	1237	Dnmt1b	1	1	1
Coprinopsis cinerea	13191	Dnmt2		1	
Dichomitus squalens	178148	Dnmt1a	2	1	1
Dichomitus squalens	98076	Dnmt1b	2	1	1
Dichomitus squalens	73086	Dnmt2		1	
Fomitiporia mediterranea	171001	Dnmt1a	2	1	

Fomitiporia mediterranea	157471	Dnmt1b	1	1	
Fomitiporia mediterranea	168758	Dnmt1b	1	1	1
Fomitiporia mediterranea	49640	Dnmt2		1	
Fomitopsis pinicola	100519	Dnmt1a	1	1	1
Fomitopsis pinicola	152255	Dnmt2		1	
Ganoderma lucidum	17911	Dnmt1a	2	1	1
Ganoderma lucidum	24739	Dnmt1b	2	2	1
Ganoderma lucidum	29617	Dnmt2		1	
Ganoderma sinense	8067	Dnmt1a	2	1	1
Ganoderma sinense	14076	Dnmt1b	2	1	1
Ganoderma sinense	7813	Dnmt2		1	
Ganoderma sp.	123613	Dnmt1a	2	1	1
Ganoderma sp.	137562	Dnmt1b		1	
Ganoderma sp.	40515	Dnmt2		1	
Laccaria bicolor	570132	Dnmt1a	1	1	1
Laccaria bicolor	306082	Dnmt1b	1	1	1
Laccaria bicolor	568876	Dnmt2		1	
Melampsora laricis-populina	85954	Masc2		1	
Neurospora crassa	2247	DIM-2	1	1	
Neurospora crassa	2034	RID		1	

Phanerochaete chrysosporium	7384	Dnmt1a		1	
Phanerochaete chrysosporium	556	Dnmt1b	1	1	
Phanerochaete chrysosporium	561	Dnmt1b		2	
Phanerochaete chrysosporium	140341	Dnmt2		1	
Postia placenta	1133669	Dnmt1a		1	1
Postia placenta	1044833	Dnmt1b	2	2	
Postia placenta	1043232	Dnmt2		1	
Puccinia graminis f. sp. tritici	3742	Masc2	1	1	
Rhodotorula graminis	52321	Masc2		1	
Schizophyllum commune	1120237	Dnmt1a		1	
Schizophyllum commune	1183828	Dnmt1a		1	1
Schizophyllum commune	1086949	Dnmt1b	1	1	1
Schizophyllum commune	1194045	Dnmt2		1	
Schizosaccharomyces pombe	19C2.02	Dnmt2		1	
Serpula lacrymans	432870	Dnmt1a	2	1	1
Serpula lacrymans	1216025	Dnmt1b	2	1	
Serpula lacrymans	1217035	Dnmt2		1	
Trametes versicolor	109093	Dnmt1a	1	1	1
Trametes versicolor	164879	Dnmt1b	1	1	1
Trametes versicolor	160146	Dnmt2		1	

Wolfiporia cocos	135973	Dnmt1a	1	1	1
Wolfiporia cocos	148860	Dnmt1b		1	

*Note: the number of Pfam domains in DNA methyltransferases. Blank cell indicates that the domain was not predicted by PfamScan. BAH domain: PF01426; DNA_methylase domain: PF00145.12; DNMT1-RFD domian: PF12047.3.

Supplementary Table S7. Potential milRNAs in *G. sinense*.

**Name**	**Length**	**Scaffold**	**Start**	**End**	**Direction**
milR-1	27	scf_6	1,871,082	1,871,108	+
milR-2	24	scf_14	1,398,018	1,398,041	+
milR-3	21	scf_1	1,613,928	1,613,948	-
milR-4	18	scf_16	326,988	327,005	+
milR-5	19	scf_19	224,943	224,961	-
milR-6	21	scf_19	836,682	836,702	-
milR-7	28	scf_2	1,479,891	1,479,918	-
milR-8	23	scf_20	158,794	158,816	+
milR-9	23	scf_20	163,764	163,786	+
milR-10	18	scf_4	1,220,625	1,220,642	+
milR-11	21	scf_4	2,791,678	2,791,698	+
milR-12	28	scf_20	178,410	178,437	-
milR-13	28	scf_20	178,718	178,745	-

milR-14	23	scf_20	178,750	178,772	-
milR-15	27	scf_6	1,043,609	1,043,635	-
milR-16	25	scf_6	1,871,124	1,871,148	+
milR-17	28	scf_20	186,489	186,516	-
milR-18	28	scf_20	186,830	186,857	-
milR-19	23	scf_20	186,862	186,884	-
milR-20	18	scf_7	809,173	809,190	+
milR-21	23	scf_7	1,045,639	1,045,661	-
milR-22	28	scf_20	193,940	193,967	-
milR-23	28	scf_20	194,281	194,308	-
milR-24	23	scf_20	194,313	194,335	-
milR-25	23	scf_20	205,466	205,488	+
milR-26	18	scf_29	15,031	15,048	+
milR-27	19	scf_8	11,795	11,813	-

Supplementary Table S8. Distribution of potential disiRNAs in *G. sinense*.

**Scaffold**	**Length**	**Start**	**End**	**Forward strand***	**Reverse strand***	**Number of chromosome**	**Location on chromosome**
scf_23	618,582	1	1,000	76	66	1	3' end
scf_23	618,582	36,001	37,000	71	74	1	3' end

scf_23	618,582	579,001	580,000	79	76	1	body
scf_23	618,582	618,001	619,000	66	76	1	body
scf_8	2,256,307	1,255,001	1,256,000	93	75	1	body
scf_2	3,546,229	832,001	833,000	170	115	2	body
scf_16	1,075,900	1,070,001	1,071,000	70	80	3	5' end
scf_7	2,513,623	2,022,001	2,023,000	62	59	3	body
scf_1	4,262,535	2,001	3,000	79	68	4	5' end
scf_37	167,957	123,001	124,000	61	72	5	5' end
scf_29	249,401	244,001	249,000	901	1089	5	3' end
scf_4	3,281,721	1	1,000	64	99	7	3' end
scf_32	208,978	4,001	5,000	102	147	7	body
scf_40	134,866	8,001	9,000	101	61	7	body
scf_40	134,866	134,001	135,000	173	263	7	5' end
scf_4	3,281,721	2,219,001	2,220,000	69	59	7	body
scf_36	168,601	1	1,000	191	176	8	5' end
scf_36	168,601	21,001	22,000	100	120	8	5' end
scf_14	1,448,948	466,001	467,000	111	93	8	body
scf_12	1,602,781	1	1,000	211	246	9	5' end
scf_12	1,602,781	11,001	12,000	209	168	9	5' end
scf_12	1,602,781	53,001	58,000	557	468	9	body

scf_43	119,835	1	3,000	851	667	10	5' end
scf_45	249,401	84,001	87,000	536	623	11	5' end
scf_38	149,579	2,001	3,000	153	97	12	body
scf_13	1,496,386	1,432,001	1,440,000	1346	1591	12	5' end
scf_47	81,581	80,001	81,000	177	260	Un	

*Number of small RNAs mapped onto forward and reverse strands of scaffolds. 5' and 3' ends were defined as the regions within 100kb from tow ends of chromosomes. Other regions were viewed as the bodies of chromosomes.

Supplementary Table S9. RNA silencing components and pathways identified in *G. sinense*.

**Gene family/name**	**Gene ID**	**Pathway**
RdRP/QDE-1 like	GS05866, GS05881	Quelling
RdRP/SAD-1 like	GS14051	MSUD
RdRP	GS00094, GS00118, GS02226, GS02328, GS06758, GS06759, GS08315, GS10773	Quelling, MSUD
Argonaute	GS03237, GS13619, GS14574, GS14719, GS14729, GS00804, GS09260, GS14571, GS14575	Quelling, MSUD
Dicer/DCL-1 like	GS00423, GS07329	Quelling, MSUD
Dicer/DCL-2 like	GS15478	Quelling

DNA helicase/QDE-3 like	GS13460	Quelling
QIP like	GS00816	Quelling, MSUD
DdRP/DNA-dependent RNA polymerase II second largest subunit	GS06515	
RPA like	GS14019	Quelling
SAD-2 like	GS11573	MSUD
SAD-3 like	GS07673, GS07762	MSUD

Supplementary Table S10. Key enzymes of MVA pathway from 25 fungal genomes.

**Species Abbr.**	**AACT[a]**	**FPS**	**HMGR**	**HMGS**	**IDI**	**LSS**	**MK**	**MVD**	**PMK**	**SE**	**SQS**
AspNi	6	2	2	2	1	3	1	1	1	1	2
BatDe	5	1	1	1	1	1	1	1	1	1	1
ConPu	4	1	1	1	1	1	1	1	1	1	1
CopCi	4	1	1	1	1	1	1	1	1	2	1
DicSq	4	5	1	1	1	1	1	1	1	1	1
FomMe	3	1	1	1	1	1	1	1	2	1	1
FomPi	3	1	1	1	1	1	1	1	1	1	1
GanLu	4	2	1	1	1	1	1	1	1	1	1
GanSi	4	2	1	1	1	1	1	1	1	1	1

GanSp	4	2	1	1	1	1	1	1	1	1	1
LacBi	4	2	1	3	1	3	1	1	1	1	1
MelLa	5	1	1	1	1	1	1	1	1	1	1
NeuCr	5	1	1	1	1	1	1	1	1	1	1
PhaCh	5	1	1	1	1	2	1	1	1[b]	1	1
PosPl	3	2	1	1	1	1	1	1	1	1	1
PucGr	4	1	1	1	1	3	1	1	2	1	1
RhoGr	6	1	1	1	1	1	1	1	1	1	1
SacCe	2	1	2	1	1	1	1	1	1	1	1
SchCo	4	1	1	1	1	1	1	1	1	2	1
SchPo	1	2	1	1	1	1	1	1	1	1	1
SerLa	4	1	1	1	1	1	1	1	1	1	1
TraVe	4	1	1	1	1	1	1	1	1	1	1
TreMe	3	1	1	1	1	1	1	1	1	1	1
UstMa	5	1	1	1	1	1	1	1	1	1	1
WolCo	4	1	1	1	1	1	1	1	1	1	1

[a]AACT: acetyl-CoA acetyltransferase; FPS: farnesyl diphosphate synthase; HMGR: 3-hydroxy-3-methyl glutaryl-CoA reductase; IDI: isopentenyl diphosphate isomerase; LSS: 2,3-oxidosqualenelanosterol cyclase; MK: mevalonate kinase; MVD: mevalonate pyrophosphate decarboxylase; PMK: phosphomevalonate kinase; SE: squalene monooxygenase; SQS: squalene synthase. [b]It was searched by comparing genome sequence with known PMK enzymes using Blast program.

Supplementary Table S11. Comparison of cytochrome P450 families in *G. sinense*, *G. lucidum*, *P. placenta* and *P. chrysosporium*.

**CYP family**	***G. sinense***	***G. lucidum***	***P. placenta***	***P. chrysosporium***
CYP51	1	2	1	1
CYP53	1	1	7	1
CYP61	1	1	1	1
CYP63	7	6	5	7
CYP502	1	1	4	1
CYP505	3	4	2	7
CYP512	31	22	14	14
CYP537	2	1	2	0
CYP642	1	1	0	0
CYP5027	0	0	9	0
CYP5035	25	16	3	13
CYP5036	0	0	0	5
CYP5037	6	6	13	5
CYP5065	1	1	0	0
CYP5136	9	7	0	5
CYP5137	1	1	6	2
CYP5138	1	1	1	1

CYP5139	8	7	8	1
CYP5140	2	1	1	1
CYP5141	2	2	4	7
CYP5142	0	0	0	7
CYP5143	0	0	0	2
CYP5144	4	3	3	34
CYP5145	0	0	0	3
CYP5146	0	0	0	6
CYP5147	0	0	0	6
CYP5148	4	2	1	2
CYP5149	0	0	1	1
CYP5150	39	36	25	6
CYP5151	1	1	1	1
CYP5152	1	1	2	2
CYP5153	0	0	0	1
CYP5154	0	0	0	1
CYP5155	0	0	0	1
CYP5156	1	1	1	1
CYP5157	0	0	0	1
CYP5158	1	1	2	1

CYP5339	0	0	2	0
CYP5340	1	2	1	0
CYP5341	1	2	3	0
CYP5342	0	0	1	0
CYP5343	0	0	1	0
CYP5344	0	0	3	0
CYP5345	0	0	1	0
CYP5346	0	0	1	0
CYP5347	1	1	2	0
CYP5348	3	3	34	0
CYP5349	1	1	2	0
CYP5350	0	0	11	0
CYP5351	1	1	1	0
CYP5352	0	0	1	0
CYP5353	0	0	1	0
CYP5354	0	0	2	0
CYP5355	0	0	1	0
CYP5356	0	0	1	0
CYP5357	2	2	0	0
CYP5358	2	1	0	0

CYP5359	53	47	0	0
CYP5360	1	1	0	0
CYP5361	1	1	0	0
CYP5362	1	1	0	0
CYP5363	0	1	0	0
CYP5364	2	3	0	0
CYP5365	1	1	0	0
CYP5366	1	1	0	0
CYP6005	2	2	0	0
Total	228	197	186	148

Supplementary Table S12. Expression level of CYP genes that were subject to DNA methylation.

**Gene ID**	**Gene name**	**Gene length**	**Mycelia***	**Methylation level**
GS11234	CYP5035N3	2,640	0	6.44
GS15687	CYP5035R5	2,376	0	83.33
GS11200	CYP5150D27a	2,590	38	70.66
GS11198	CYP5150D27b	2,753	329	20.34
GS11205	CYP5150D27c	2,522	0	58.68

GS08700	CYP5359A1	2,066	0	159.24
GS11357	CYP5359E4	2,231	0	54.24

*indicates FPKM values of expression.

Supplementary Table S13. Classification of transcription factor families in *G. sinense*.

Family	No. of genes	Family	No. of genes	Family	No. of genes
Aft1	1	Heteromeric CCAAT factors	7	SPT4	1
APSES	5	HLH	10	ssDNA-binding transcriptional regulator	1
AT-hook	5	HMG	23	TEA/ATTS	4
AT-rich interaction region	2	Homeobox	14	Transcription factor jumonji	11
bHLH	11	Homeodomain-like	34	Transcription factor TFIIS	4
Bromodomain transcription factor	2	HORMA	3	TUP1	1
bZIP	15	HTH	1	Winged helix repressor DNA-binding	28
C2H2 zinc finger	65	MADS-box	2	YL1 nuclear protein	1
CCR4-Not complex component, Not1	1	Myb	11	Zinc finger, CCHC-type	75

Copper	4	NusA	1	Zinc finger, DHHC-type	4
Cyclin	3	p53-like transcription factor	2	Zinc finger, GRF-type	2
DDT	1	PHD	21	Zinc finger, MIZ-type	1
Forkhead	3	RFX DNA-binding domain	1	Zinc finger, NF-X1-type	1
GATA type zinc finger	11	SART1	1	Zinc finger, PARP-type	2
Helix-turn-helix type 3	2	SET	19	Zinc finger, Rad18-type putative	2
Helix-turn-helix, Psq	1	SGT1	1	Zn2Cys6	85

Supplementary Table S14. Classification of transporters in *G. sinense*. Transporters were classified based on transporter classification system (TC).

**TC number**	**TC name**	**Number of genes**
1.A.1	The Voltage-gated Ion Channel (VIC) Superfamily	11
1.A.11	The Ammonia Transporter Channel (Amt) Family	2
1.A.12	The Intracellular Chloride Channel (CLIC) Family	7
1.A.15	The Non-selective Cation Channel-2 (NSCC2) Family	1
1.A.16	The Formate-Nitrite Transporter (FNT) Family	1
1.A.17	The Calcium-Dependent Chloride Channel (Ca-ClC) Family	1
1.A.23	The Small Conductance Mechanosensitive Ion Channel (MscS) Family	2
1.A.24	The Gap Junction-forming Connexin (Connexin) Family	1

1.A.26	The Mg2+ Transporter-E (MgtE) Family	71
1.A.30	The H+- or Na+-translocating Bacterial Flagellar Motor/ExbBD Outer Membrane Transport Energizer (Mot/Exb) Superfamily	1
1.A.33	The Cation Channel-forming Heat Shock Protein-70 (Hsp70) Family	20
1.A.35	The CorA Metal Ion Transporter (MIT) Family	4
1.A.38	The Golgi pH Regulator (GPHR) Family	1
1.A.4	The Transient Receptor Potential Ca2+ Channel (TRP-CC) Family	8
1.A.47	The Nucleotide-sensitive Anion-selective Channel, ICln (ICln) Family	1
1.A.51	The Voltage-gated Proton Channel (VPC) Family	1
1.A.56	The Copper Transporter (Ctr) Family	4
1.A.6	The Epithelial Na+ Channel (ENaC) Family	1
1.A.8	The Major Intrinsic Protein (MIP) Family	10
1.A.9	The Neurotransmitter Receptor, Cys loop, Ligand-gated Ion Channel (LIC) Family	1
1.B.12	The Autotransporter-1 (AT-1) Family	13
1.B.33	The Outer Membrane Protein Insertion Porin (Bam Complex) (OmpIP) Family	2
1.B.8	The Mitochondrial and Plastid Porin (MPP) Family	1
1.C.33	The Cathelicidin (Cathelicidin) Family	1
1.C.39	The Membrane Attack Complex/Perforin (MACPF) Family	2
1.C.57	The Clostridial Cytotoxin (CCT) Family	1
1.C.63	The α-Latrotoxin (Latrotoxin) Family	2
1.C.71	The Cytolytic Delta Endotoxin (Cyt1/2) Family	7
1.C.82	The Pore-forming Amphipathic Helical Peptide HP(2-20) (HP2-20) Family	1
1.F.1	The Synaptosomal Vesicle Fusion Pore (SVF-Pore) Family	8
1.G.7	The Reovirus FAST Fusion Protein (R-FAST) Famly	1
2.A.1	The Major Facilitator Superfamily (MFS)	235

2.A.100	The Ferroportin (Fpn) Family	1
2.A.16	The Telurite-resistance/Dicarboxylate Transporter (TDT) Family	11
2.A.17	The Proton-dependent Oligopeptide Transporter (POT) Family	1
2.A.18	The Amino Acid/Auxin Permease (AAAP) Family	5
2.A.19	The Ca2+	8
2.A.2	The Glycoside-Pentoside-Hexuronide (GPH)	3
2.A.21	The Solute	4
2.A.29	The Mitochondrial Carrier (MC) Family	39
2.A.3	The Amino Acid-Polyamine-Organocation (APC) Family	25
2.A.31	The Anion Exchanger (AE) Family	1
2.A.36	The Monovalent Cation	5
2.A.37	The Monovalent Cation	2
2.A.38	The K+ Transporter (Trk) Family	3
2.A.39	The Nucleobase	10
2.A.4	The Cation Diffusion Facilitator (CDF) Family	4
2.A.40	The Nucleobase	1
2.A.41	The Concentrative Nucleoside Transporter (CNT) Family	1
2.A.43	The Lysosomal Cystine Transporter (LCT) Family	1
2.A.44		1
2.A.47	The Divalent Anion	2
2.A.49	The Chloride Carrier/Channel (ClC) Family	3
2.A.5	The Zinc (Zn2+)-Iron (Fe2+) Permease (ZIP) Family	4
2.A.50	The Glycerol Uptake (GUP) Family	1
2.A.52	The Ni2+-Co2+ Transporter (NiCoT) Family	1
2.A.53	The Sulfate Permease (SulP) Family	6
2.A.55	The Metal Ion (Mn2+-iron) Transporter (Nramp) Family	3
2.A.57	The Equilibrative Nucleoside Transporter (ENT) Family	1

2.A.59	The Arsenical Resistance-3 (ACR3) Family	1
2.A.6	The Resistance-Nodulation-Cell Division (RND) Superfamily	8
2.A.66	The Multidrug/Oligosaccharidyl-lipid/Polysaccharide (MOP) Flippase Superfamily	6
2.A.67	The Oligopeptide Transporter (OPT) Family	14
2.A.7	The Drug/Metabolite Transporter (DMT) Superfamily	29
2.A.82	The Organic Solute Transporter (OST) Family	1
2.A.85	The Aromatic Acid Exporter (ArAE) Family	2
2.A.89	The Vacuolar Iron Transporter (VIT) Family	1
2.A.9	The Cytochrome Oxidase Biogenesis (Oxa1) Family	1
2.A.92	The Choline Transporter-like (CTL) Family	1
2.A.94	The Phosphate Permease (Pho1) Family	2
2.A.96	The YaaH (YaaH) Family	1
2.A.97	The Mitochondrial Inner Membrane K+/H+ and Ca2+/H+ Exchanger (LetM1) Family	2
3.A.1	The ATP-binding Cassette (ABC) Superfamily	53
3.A.16	The Endoplasmic Reticular Retrotranslocon (ER-RT) Family	25
3.A.17	The Phage T7 Injectisome (T7 Injectisome) Family	1
3.A.18	The Nuclear mRNA Exporter (mRNA-E) Family	46
3.A.19	The TMS Recognition/Insertion Complex (TRC) Family	1
3.A.2	The H+- or Na+-translocating F-type, V-type and A-type ATPase (F-ATPase) Superfamily	20
3.A.20	The Peroxisomal Protein Importer (PPI) Family	99
3.A.3	The P-type ATPase (P-ATPase) Superfamily	21
3.A.4	The Arsenite-Antimonite (ArsAB) Efflux Family	1
3.A.5	The General Secretory Pathway (Sec) Family	29
3.A.7	The Type IV (Conjugal DNA-Protein Transfer or VirB) Secretory Pathway (IVSP) Family	2
3.A.8	The Mitochondrial Protein Translocase (MPT) Family	22

3.A.9	The Chloroplast Envelope Protein Translocase (CEPT or Tic-Toc) Family	20
3.B.1	The Na+-transporting Carboxylic Acid Decarboxylase (NaT-DC) Family	5
3.D.1	The H+ or Na+-translocating NADH Dehydrogenase (NDH) Family	24
3.D.2	The Proton-translocating Transhydrogenase (PTH) Family	1
3.D.3	The Proton-translocating Quinol	1
3.D.4	The Proton-translocating Cytochrome Oxidase (COX) Superfamily	11
3.D.5	The Na+-translocating NADH	2
3.E.1	The Ion-translocating Microbial Rhodopsin (MR) Family	3
4.C.1	The Proposed Fatty Acid Transporter (FAT) Family	19
4.C.2	The Carnitine O-Acyl Transferase (CrAT) Family	4
4.C.3	The Acyl-CoA Thioesterase (AcoT) Family	1
5.A.4		4
5.B.1	The Phagocyte (gp91phox) NADPH Oxidase Family	12
8.A.13	The Tetratricopeptide Repeat (Tpr1) Family	1
8.A.15	The K+ Channel Accessory Protein (KChAP) Family	1
8.A.21	The Stomatin/Podocin/Band 7/Nephrosis.2/SPFH (Stomatin) Family	1
8.A.25	The Ezrin/Radixin/Moesin (Ezrin) Family	1
8.A.27	The Phospholipid Importer β-subunit (PLI-β) Family	2
8.A.28	The Ankyrin (Ankyrin) Family	9
8.A.30	The Nedd4-Family Interacting Protein-2 (Nedd4) Family	4
8.A.32	The β-Amyloid Cleaving Enzyme (BACE1) Family	32
8.A.34	The Endophilin (Endophilin) Family	8
8.A.5	The Voltage-gated K+ Channel β-subunit (Kvβ) Family	36
8.A.6	The Auxiliary Nutrient Transporter (ANT) Family	1
8.B.9	The Triflin Toxin (Triflin or CRISP) Family	8
9.A.1	The Non ABC Multidrug Exporter (N-MDE) Family	10

9.A.10		20
9.A.14	The G-protein-coupled receptor (GPCR) Family	33
9.A.17	The Integral Membrane Peroxisomal Protein Importer-2 (PPI2) Family	3
9.A.19	The Lipid Intermediate Transporter (Arv1) Family	1
9.A.2	The Endomembrane protein-70 (EMP70) Family	1
9.A.26	The Lipid-translocating Exporter (LTE) Family	5
9.A.34		2
9.A.36	The Ca2+-dependent Phospholipid Scramblase (Scramblase) Family	1
9.A.40	The HlyC/CorC (HCC) Family	3
9.A.45	The Magnesium Transporter1 (MagT1) Family	1
9.A.48	The Unconventional Protein Secretion (UPS) System	2
9.A.50	The Nuclear t-RNA exporter (t-Exporter) Family	26
9.A.55	The TMEM205 (TMEM205) Family	1
9.A.6	The ATP Exporter (ATP-E) Family	3
9.A.8	The Ferrous Iron Uptake (FeoB) Family	1
9.B.1	The Integral Membrane CAAX Protease (CAAX Protease) Family	2
9.B.10	The Putative Tripartite Zn2+ Transporter (TZT) Family	2
9.B.102	The YedE/YeeE (YedE/YeeE) Family	1
9.B.103	The Putative Ca2+ Uniporter (GC1qR) Family	1
9.B.12	The (Salt or Low Temperature) Stress-induced Hydrophobic Peptide (SHP) Family	2
9.B.17	The VAMP-associated protein (VAP) Family	1
9.B.21	The Frataxin (Frataxin) Family	1
9.B.24	The DUF805 or PF05656 (DUF805) Family	2
9.B.25	The Mitochondrial Inner/Outer Membrane Fusion (MMF) Family	13
9.B.26		1
9.B.27	The DedA or YdjX-Z (DedA) Family	2

9.B.32	The DUF3302 or Pfam11742 (YibI) Family	4
9.B.35	The Putative Thyronine-Transporting Transthyretin (Transthyretin) Family	1
9.B.37	The Huntington-interacting Protein 14 (HIP14) Family	6
9.B.38	The Myelin Proteolipid Protein (MPLP) Family	1
9.B.45	The Arg/Asp/Asp (RDD) Family	28
9.B.7	The Putative Sulfate Transporter (CysZ) Family	1
9.B.71	The Camphor Resistance (CrcB) Family	1
9.B.82	Endoplasmic Reticulum Retrieval Protein1 (Putative Heavy Metal Transporter) (Rer1) Family	1
9.B.87	The Selenoprotein P Receptor (SelP-receptor) Family	1
